# Gut microbiome mediates the protective effects of exercise after myocardial infarction

**DOI:** 10.1186/s40168-022-01271-6

**Published:** 2022-05-31

**Authors:** Qiulian Zhou, Jiali Deng, Xue Pan, Danni Meng, Yujiao Zhu, Yuzheng Bai, Chao Shi, Yi Duan, Tianhui Wang, Xinli Li, Joost PG Sluijter, Junjie Xiao

**Affiliations:** 1 Institute of Geriatrics (Shanghai University), (The Sixth People’s Hospital of Nantong), School of Medicine, Affiliated Nantong Hospital of Shanghai University, Shanghai University, Nantong, 226011 China; 2 Cardiac Regeneration and Ageing Lab, School of Life Science, Institute of Cardiovascular Sciences, Shanghai Engineering Research Center of Organ Repair, Shanghai University, Shanghai, 200444 China; 3 Department of Cardiology, The First Affiliated Hospital of Nanjing Medical University, Nanjing, 210029 China; 4 Department of Cardiology, Laboratory of Experimental Cardiology, University Utrecht, University Medical Center Utrecht, 3584 CX Utrecht, The Netherlands; 5 UMC Utrecht Regenerative Medicine Center, University Medical Center Utrecht, 3508 GA Utrecht, The Netherlands

**Keywords:** Exercise, Myocardial infarction, Gut microbiome, Metabolites, NRF2

## Abstract

**Background:**

Gut microbiota plays important roles in health maintenance and diseases. Physical exercise has been demonstrated to be able to modulate gut microbiota. However, the potential role of gut microbiome in exercise protection to myocardial infarction (MI) remains unclear.

**Results:**

Here, we discovered exercise training ameliorated cardiac dysfunction and changed gut microbial richness and community structure post-MI. Moreover, gut microbiota pre-depletion abolished the protective effects of exercise training in MI mice. Furthermore, mice receiving microbiota transplants from exercised MI mice had better cardiac function compared to mice receiving microbiota transplants from non-exercised MI mice. Mechanistically, we analyzed metabolomics in fecal samples from exercised mice post-MI and identified 3-Hydroxyphenylacetic acid (3-HPA) and 4-Hydroxybenzoic acid (4-HBA), which could be applied individually to protect cardiac dysfunction post-MI and apoptosis through NRF2.

**Conclusions:**

Together, our study provides new insights into the role of gut microbiome in exercise protection to MI, offers opportunities to modulate cardiovascular diseases by exercise, microbiome and gut microbiota-derived 3-HPA and 4-HBA.

Video Abstract

**Supplementary Information:**

The online version contains supplementary material available at 10.1186/s40168-022-01271-6.

## Background

The number of genes associated with gut microbes vastly exceed the total complement of genes in the host [1]. The gut microbiome is critical for maintaining host physiology and homeostasis through metabolic exchange and co-metabolism of substrates [2]. Moreover, the gut microbiome has emerged as an important regulator in numerous facets of human health and disease. Mounting evidence has linked alterations of gut microbiome with a variety of disease including obesity, type 2 diabetes mellitus, fatty liver, hypertension, heart failure, and myocardial infarction (MI) [3–9]. As the gut microbiome is therapeutically modifiable, manipulation represents a novel opportunity to combat chronic diseases.

MI is a major cause of death worldwide [10]. With the advances in therapeutic approaches of mechanical reperfusion such as percutaneous coronary intervention (PCI), the acute mortality rates of MI have been significantly reduced while the post-infarction cardiac remodeling and heart failure has been a huge healthcare and economic burden [11]. Cardiac rehabilitation (CR) is a recommended effective adjunct therapy for patients of MI and exercise training is a powerful tool in CR programs [12, 13]. Exercise-based CR can lead to improved exercise capacity and prognosis in MI patients [14]. Understanding the molecular mechanism underlying the protective effects of exercise in MI can help identify novel therapy for post-infarction cardiac remodeling and heart failure.

Alterations of the gut microbiome have been reported upon MI in both animal models and human patients [15, 16]. Mice receiving exercise training demonstrated favorable changes in the composition of gut microbiota [17]. In humans, athletes have a higher richness and diversity of the gut microbiome [18]. However, the role of gut microbiome in the protective effects of exercise in cardiac dysfunction post-MI is unclear. In our study, we aimed to explore the potential roles of gut microbiome in exercise protection in MI.

## Methods

### Animals

Male C57BL/6 mice aged 8–10 weeks were purchased from Cavens Lab Animal (Changzhou, China), and raised at the specific pathogen-free (SPF) laboratory animal facility of Shanghai University (Shanghai, China). Mice were maintained on a 12-h light/dark cycle at 25 °C and provided free access to commercial rodent chow (sterilized by Cobalt-60) and tap water (high-temperature sterilization) before initiation of the experiments. Randomized grouping was used and the same group of mice were co-housed with less than 5 animals per cage. All animal experiments were in accordance with the guidelines approved by the committee on the Ethics of Animal Experiments of Shanghai University.

Left anterior descending coronary artery was ligated to create the MI mice model, as we applied before [19]. Under sterile conditions, left anterior descending coronary artery was tied by a 7–0 silk suture. Sham-operated (Sham) mice were treated with the same surgery without tying the left anterior descending coronary artery. Exercise therapy via running was carried out 1-week after MI surgery.

In the running model, MI mice were placed in a treadmill, starting from 10 min with 5 m per minute and we increased distance 2 m per minute each day until 60 min at 15 m per minute [20]. Mice were sacrificed after training a total of 8 weeks of running.

The treadmill running test was used to measure endurance capacities of mice. Mice were placed in the treadmill to adapt to the environment for 5 min and then run at the speed of 15 m/min. We increased the speed by 1 m/min every 4 min and recorded the running speed and running time when the mice were exhausted. According to the speed and time, the general movement path of mice was calculated as endurance capacities.

Transthoracic echocardiography examination was used to determine cardiac function as demonstrated by left ventricular ejection fraction (EF) and fractional shortening (FS) performed with the VisualSonics Vevo 2100system (VisualSonics Inc, Toronto, Ontario, Canada) with a 30 MHz central frequency scan head and measured from M-mode images taken from the parasternal short-axis view at papillary muscle level. Each mouse was anesthetized with 1.5% isoflurane and measured at least three times.

### 16S rDNA profiling of gut microbiota in mice

At the endpoint of the experiments, one mouse was put in a cage and then the mice feces (100 mg per mice) collected under sterile conditions were frozen using liquid nitrogen and stored at – 80 ℃. Fecal samples, packed with dry ice, were sent for analyses to the laboratory at Majorbio Bio-Pharm Technology Co., Ltd. (Shanghai, China).

The total genomic DNA was isolated from mice feces samples using the QIAGEN QIAamp Fast DNA Stool Mini Kit, according to manufacturer’s instructions. After checked on 1% agarose gel and determined with Nano-Drop 2000 UV–vis spectrophotometer, the hypervariable region V3-V4 of the bacterial 16S rRNA gene were amplified by an ABI GeneAmp® 9700 PCR thermocycler. The primer pairs used: 338 forward primer (5′-ACTCCTACGGGAGGCAGCAG-3′) and 806 reverse primer (5′-GGACTACHVGGGTWTCTAAT-3′). The PCR reactions were performed in triplicate according: 5 × TransStart FastPfu buffer 4 μL, 2.5 mM dNTPs 2 μL, forward primer (5 μM) 0.8 μL, reverse primer (5 μM) 0.8 μL, TransStart FastPfu DNA Polymerase 0.4 μL, template DNA 10 ng, and finally ddH_2_O up to 20 μL. The PCR conditions were as follows: 95 °C for 3 min, followed by 27 cycles of 95 °C for 30 s, 55 °C for 30 s, 72 °C for 45 s, and an extension of 72 °C for 10 min. All PCR products were extracted from agarose gels (2% in TAE buffer) and purified with AxyPrep DNA Gel Extraction Kit (Axygen Biosciences, USA), and quantified using a QuantiFluor™-ST fluorescent quantitative system (Promega, USA).

Purified amplicons were constructed and paired-end sequencing was performed on an Illumina MiSeq platform (Illumina, USA) by Majorbio Bio-Pharm Technology Co. Ltd. (Shanghai, China). The raw 16S rRNA gene sequencing reads were demultiplexed and quality-filtered using fastp (version 0.20.0) [21], then merged by FLASH (version 1.2.7) [22]. Then the high-quality sequences were de-noised using DADA2 plugin in the Qiime2 (version 2020.2) pipeline with recommended parameters, which obtains single nucleotide resolution based on error profiles within samples. Taxonomic assignment of amplicon sequence variants (ASVs) was performed using the Naive bayes consensus taxonomy classifier implemented in Qiime2 and the SILVA 16S rRNA database (v138). Analyses of the 16S rRNA microbiome sequencing data was performed using the free online platform of Majorbio Cloud Platform (cloud.majorbio.com).

For 16S rRNA statistical analyses, Pan was calculated by Usearch. Alpha diversity was calculated by Mothur. Principal co-ordinates analysis (PCoA) and non-metric multidimensional scaling analysis (NMDS) were performed by R version 3.3.1, vegan, and mixOmics package, and significant differences were assessed by analysis of similarities (ANOSIM). Enterotype analysis was calculated by the statistical clustering method, which was based on Jensen-Shannon distance (JSD). Correlation analysis was assessed by Spearman. Heatmap, barplot, and pielot were performed by R version 3.3.1, vegan, and mixOmics package. Significant differences were assessed by Wilcoxon rank-sum with FDR.

### Antibiotic treatment and fecal microbiota transplantation

The maximal dose of antibiotic cocktail was prepared by mixing ampicillin (0.25 mg/mL), metronidazole (0.25 mg/mL), neomycin (0.25 mg/mL), and vancomycin (0.125 mg/mL) in autoclaved water [15]. Animals that received the antibiotic cocktail were referred to as ABX mice, whereas drink autoclaved water were referred to as untreated mice. ABX mice drank maximal dose of antibiotic cocktail for 7 days post-MI surgery, followed by normal autoclaved water. 1/4 ABX mice received a 25% dose of the antibiotic cocktail until the end of the experiment.

For microbiota transplantation, the endpoint-mice fecal samples (100 mg per mice), collected under sterile conditions, were resuspended with pre-cooled PBS, centrifuged for supernatant with 1000 rpm for 5 min at 4 °C, and this step was repeated twice. Then glycerin (20%) was added before storage at – 80 ℃. Mice drank maximal dose of antibiotic cocktail for 7 days post-MI surgery and then were orally inoculated (200 μL for each mouse) at 1-day interval for 1 week. Cardiac function and histopathological detection were detected after 8 weeks.

### MicrobioMET

At the endpoint of the experiments, one mouse was put in a cage and then feces samples (100 mg per mice) collected under sterile conditions were frozen using liquid nitrogen and stored at – 80 ℃. Fecal samples packed with dry ice were sent to the laboratory at Metabo-Profile Biotechnology Co., Ltd. (Shanghai, China). Metabo-Profile lnc. (Shanghai, China) performed the quantitation of bacterial metabolites as previously reported [23, 24], using a database composed of 132 standards obtained from Sigma-Aldrich (St. Louis, MO, USA), Steraloids Inc. (Newport, RI, USA), and TRC Chemicals (Toronto, ON, Canada). In this project, the microbial metabolites were quantitated by ultra-performance liquid chromatography coupled to tandem mass spectrometry (UPLC-MS/MS) system (ACQUITY UPLC-Xevo TQ-S, Waters Corp., Milford, MA, USA).

Univariate statistical analyses were assessed either by student T test or Wilcoxon Test, depending on the normality of data and homogeneity of variance. Multivariate statistical analyses (OPLS-DA) were used to observe the similarities and dissimilarities among the groups. Pathway bubbleplot and network diagram were assessed by R language, ggplot2 package, and igragh and Cairo packets. Correlation analysis was assessed by Spearman.

### Metabolites treatment

To prepare the metabolites supplement, 3-Hydroxyphenylacetic acid (3-HPA, SANTA CRUZ), 4-Hydroxybenzoic acid (4-HBA, SIGMA), and p-Hydroxyphenylacetic acid (4-HPA, SIGMA) were individually dissolved in autoclaved in saline. Mice drank maximal dose of the antibiotic cocktail for 7 days post-MI surgery and then were orally inoculated (3-HPA, 4-HBA, 4-HPA, 6, 25 mg/kg per day) at 1-day interval for 4 weeks.

### Histological analysis

Histopathological detection was used to analyze ventricular remodeling. Hematoxylin–Eosin (HE) staining and wheat germ agglutinin (WGA) staining was used to measure cardiomyocyte size. For HE staining, mouse heart tissue fixed in 4% paraformaldehyde followed by buried with paraffin. Paraffin Sect. (5 μm) were treated with Xylene and Ethanol separately. Section incubated with Hematoxylin staining and Eosin staining gradually and 30–60 fields per Sect. (400 × magnification) were viewed under microscope. For WGA staining, mouse heart tissue snappily frozen in OCT at – 80 ℃. Frozen Sects. (10 μm) were fixed in 4% paraformaldehyde followed by washing with PBS. Section incubated with WGA-FITC (1:100, sigma # L4895) and DAPI gradually and 30–60 fields per Sect. (400 × magnification) were viewed. Cell size was measured with ImageJ.

Cardiac fibrosis was evaluated by Masson's Trichrome staining. For Masson’s Trichrome staining, mouse heart tissues were fixed and treated with same way of paraffin section. Then section successively incubated with iron hematoxylin staining, ponceau acid magenta, phosphomolybdate staining, and aniline blue dyeing. Then glycerin sealed section and 20 fields per Sect. (200 × magnification) were viewed. Fibrosis was measured with ImageJ.

### Cell isolation, culture, treatments, and immunofluorescent staining

Neonatal rat cardiomyocytes (NRCMs) were isolated and cultured as previously reported [25, 26]. To induce OGD/R, NRCMs were firstly cultured for 8 h with serum-free no glucose DMEM (Gibco, U.S.A) in an air-tight chamber with a humidified hypoxic atmosphere containing 5% CO_2_ and 95% N_2_ at 37 °C. After exposure to oxygen glucose deprivation for 8 h, the culture medium was replaced with serum and glucose-containing DMEM and transferred to a normal incubator for recovery for 12 h. For metabolites treatment, 3-HPA and 4-HBA were incubated with indicated duration and concentration. Terminal deoxynucleotidyl transferase-mediated dUTP in situ nick-end-labeling (TUNEL) staining was conducted to detect apoptotic nuclei by confocal microscopy in α-actinin-labeled cardiomyocytes, as described before [27]. Fifteen fields/sample (200 × magnification) were viewed under a confocal microscope (Leica, Germany).

### RNA isolation and relative quantitative RT-PCR

RNA isolation and relative quantification RT-PCR were performed as described previously [26]. The sequences of primers used for RT-PCR were as follows. ANP forward: AGCCGTTCGAGAACTTGTCTT, ANP reward: CAGGTTATTGCCACTT AGGTTCA, BNP forward: GAGTCCTTCGGTCTCAAG GC, BNP reward: TACAG CCCAAACGACTGACG. 18 s RNA were used as internal controls.

### Western blotting

Mouse heart tissue were lysed by RIPA lysis buffer (Keygen Biotech) complemented with phenylmethylsulfonyl fluoride (PMSF). BCA Protein Assay (TaKaRa) was adopted to determine protein concentrations. After equal quantities, total proteins were separated in SDS-PAGE gel (10–12%). Using wet tank transfer method, protein bands were transferred onto PVDF membranes (PALL). After blocked with 5% BSA and washed with TBST, protein bands were blotted with primary antibodies at 4 °C overnight as follows: Bax (Abclonal, A12009, 1:1000 dilution), Bcl_2_ (affbiotech, AF6139, 1:1000 dilution), Caspase3 (Abclonal, A2156, 1:1000 dilution), COL1A2 (Bioworld, BS1530, 1:1000 dilution), NRF2 (Abclonal, A0674, 1:1000 dilution), and β-actin (Bioworld, AP0060, 1:10,000 dilution). After washing with TBST, protein bands were blotted with HRP conjugated secondary antibody and monitored using ECL buffer (Tanon). Quantifications of Western Blots were measured with ImageJ.

### Statistical analysis

Data from the mouse and cell model were expressed as mean ± SD. Significant differences were assessed either by two-tailed student *t* test, one-way ANOVA followed by Bonferroni’s post hoc test, or two-way ANOVA followed by Bonferroni’s post hoc test when appropriate. *P* values less than 0.05 were statistically different. Analyses were performed using GraphPad Prism 7.0.4.

### Data availability

The raw reads were deposited into the NCBI Sequence Read Archive (SRA) database (Accession Number: SRP287461). The data for this study were available by contacting the corresponding author upon reasonable request.

## Results

### Exercise training mitigates cardiac dysfunction post-MI

**Fig. 1:**
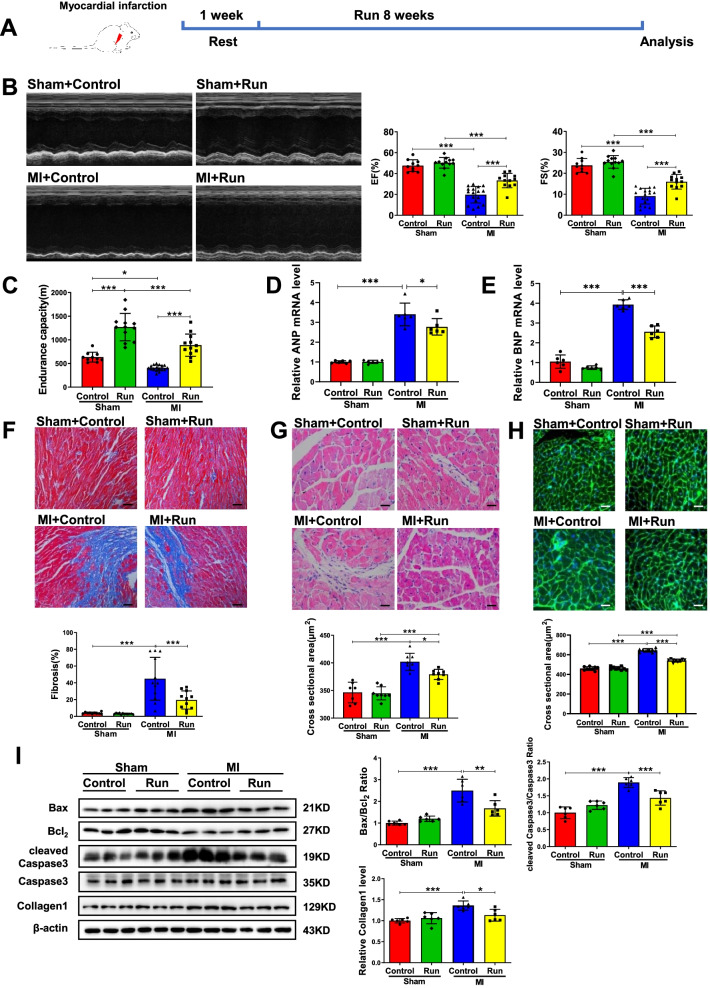
Running training protects against cardiac dysfunction after MI. **A** The schedule of running training after MI. **B** Running increased ejection fractions (EF) and fractional shortening (FS) post-MI (*n* = 10:12:17:12). **C** Running increased endurance capacity post-MI (*n* = 10:12:17:12). **D**, **E** Running decreased cardiac ANP and BNP mRNA expression post-MI (*n* = 6:6:6:6). **F** Running decreased cardiac fibrosis post-MI (*n* = 10:12:12:11). **G** Running decreased cardiac cross sectional area post-MI by H&E (*n* = 7:8:8:8). **H** Running decreased cardiac cross sectional area post-MI by WGA (*n* = 9:9:9:9). **I** Running decreased cardiac Bax/Bcl_2_, cleaved Caspase3/Caspase3 and Collagen1 post-MI (*n* = 6:6:6:6). Scale bar: 50 μm in F and 25 μm in G and H. Data were represented as mean ± SD. Significant differences were assessed by two-way ANOVA followed by Bonferroni’s multiple comparisons test. *p < 0.05, **p < 0.01, ***p < 0.001

To determine if exercise training could be protective for cardiac dysfunction post-MI, mice were subjected to treadmill running 1-week post-MI (Fig. 1A). Eight-week exercise training significantly improved cardiac function as demonstrated by elevated ejection fractions (EF) and fractional shortening (FS) (Fig. 1B) in post-MI mice. Moreover, endurance capacity of these mice was also enhanced (Fig. 1C), whereas the mRNA expression levels of atrial natriuretic peptide (ANP) and brain natriuretic peptide (BNP) were increased in MI and were decreased by exercise training (Fig. 1D and E). Moreover, Masson staining showed that exercise training could significantly reduce post-MI cardiac fibrosis (Fig. 1F), while hematoxylin–eosin (HE) and wheat germ agglutinin (WGA) staining displayed that the ultrastructure of myocardium was well-preserved and cardiomyocyte cross-sectional area was decreased in the exercised group post-MI (Fig. 1G and H). Additionally, the protein expression levels of Collagen1, and the ratios of Bax/Bcl_2_ and cleaved Caspase3/Caspase3 were increased in MI and were decreased by exercise training (Fig. 1I). Collectively, these data consistently demonstrate that exercise training can mitigate cardiac dysfunction post-MI.

### Exercise training changes gut microbial richness and community structure post-MI

To identify whether the protective effects of exercise training for MI are associated with gut microbial content and composition, we performed 16S rDNA profiling of fecal samples from four groups, including Sham + control, MI + control, Sham + Run, and MI + Run. Bacterial DNA was extracted from fecal samples, sequenced on the Illumina platform, and an average of 56,638.65 ± 12,105.23 (SD) sequences per sample were generated (Additional file 1: Table S1).

**Fig. 2:**
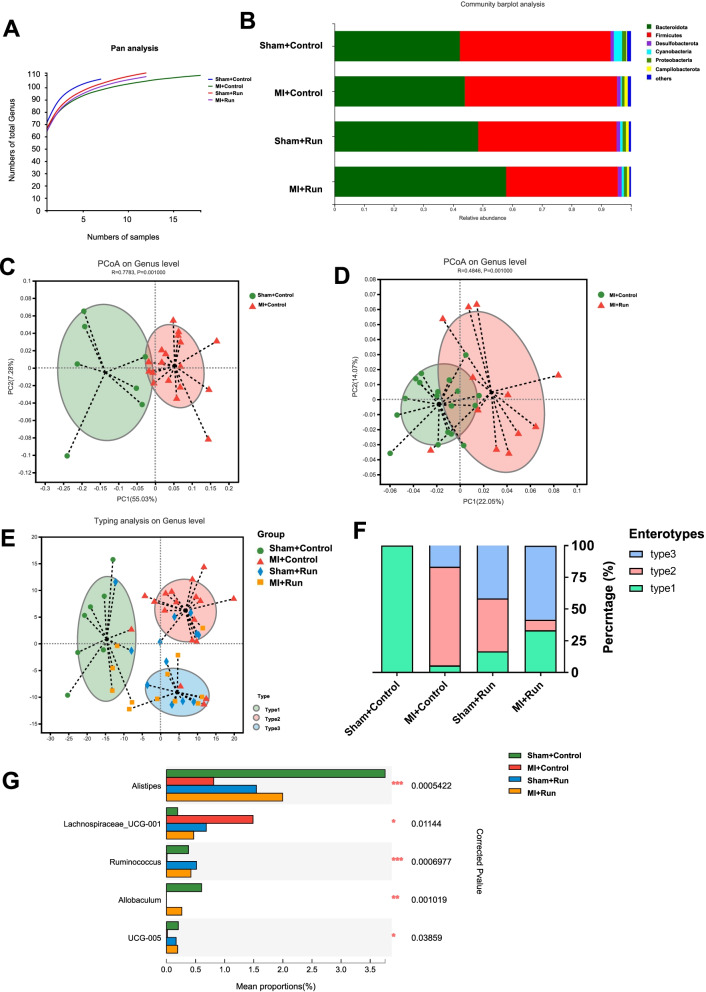
Running training changes gut microbial richness and community structure in mice after MI. **A** Pan analysis for four groups based on genus level (*n* = 7:18:12:12). **B** Fecal bacterial community at the phylum level among Sham + control, MI + control, Sham + Run, and MI + Run. **C** PCoA analysis based on the relative abundance of genus between Sham + control and MI + control groups. **D** PCoA analysis based on the relative abundance of genus between MI + control and MI + Run groups. **E** A total of 49 samples were clustered into enterotype 1 (green), enterotype 2 (red), and enterotype 3 (blue) at the genus level. **F** The percentage of Sham + control, MI + control, Sham + Run, and MI + Run samples distributed in three enterotypes. **G** Five changed genera among Sham + control, MI + control, Sham + Run, and MI + Run. Significant differences were assessed by ANOSIM analysis in **C**, **D**; abund jaccard analysis in **E**; Wilcoxon rank-sum with FDR in **F**. *p < 0.05, **p < 0.01, ***p < 0.001

Pan analysis based on the genus level indicated that MI decreased the level of gene enrichment while running exercise rescued the decreased gene enrichment in MI (Fig. 2A). Comparison of alpha diversity (the observed richness) based on the genus level indicated that MI and running did not change the community diversity of gut microbial (Additional file 1: Table S2 and S3).

In the feces of these 49 mice, Bacteroidota was predominant, represented by 42.30–57.92% of the 16S rRNA gene sequences. The second most abundant phylum was Firmicutes, with 37.68–50.83% in each group. Interestingly, in MI + Run group, Bacteroidota was higher abundant and Firmicutes was lower abundant than in the non-exercise-group (Fig. 2B and S1, Additional file 1: Table S4). At the genus level, detected ASVs were distributed among 136 different bacterial genera in total (Fig. S2 and S3, Additional file 1: Table S5). We used principal coordinate analysis (PCoA) to examine the community structures of the gut microbiotas, and found that the gut microbiota of mice in MI were separated from Sham (Fig. 2C). In addition, the gut microbiota of MI mice in the running group were separated from sedentary group (Fig. 2D).

Subsequently, genus level was performed to cluster the 49 samples into two distinct enterotypes by using JSD analysis. The major contributors in enterotype 1 (green) were *Muribaculaceae* (28.40%) and* Lachnospiraceae* (14.51%), in enterotype 2 (red) were *Lachnospiraceae* (19.45%) and *Muribaculaceae* (17.99%), and enterotype 3 (blue) were *Muribaculaceae* (26.40%) and *Prevotellaceae* (18.78%) (Fig. 2E). The percentage of enterotype 1 was 100% in Sham + control group, while enterotype 2 (77.78%) was more prevalent in MI + control group. Interestingly, enterotype 3 (58.33%) and enterotype 1 (33.33%) were more prevalent in MI + Run group (Fig. 2E and F). At the genus level, we found the abundance of 5 genera was changed in exercise after MI, namely *Alistipes*, *Lachnospiraceae_UCG-001*, *Ruminococcus*, *Allobaculum*, and *Oscillospiraceae UCG-005* (Fig. 2G, Additional file 1: Table S6 and S7). These findings suggest that running in MI may change the gut microbiota community structure.

To identify whether the change in gut microbial are associated with cardiac function parameters, we analyzed the relationship between different cardiac function indexes and the top 50 genera. *Alistipes* and *Ruminococcus* were most positively correlated with EF and FS, while *Lachnospiraceae_UCG-001* was most negatively correlated with EF and FS (Fig. S4).

### Antibiotic treatment inhibits the protective effects of exercise training in cardiac dysfunction post-MI

**Fig. 3:**
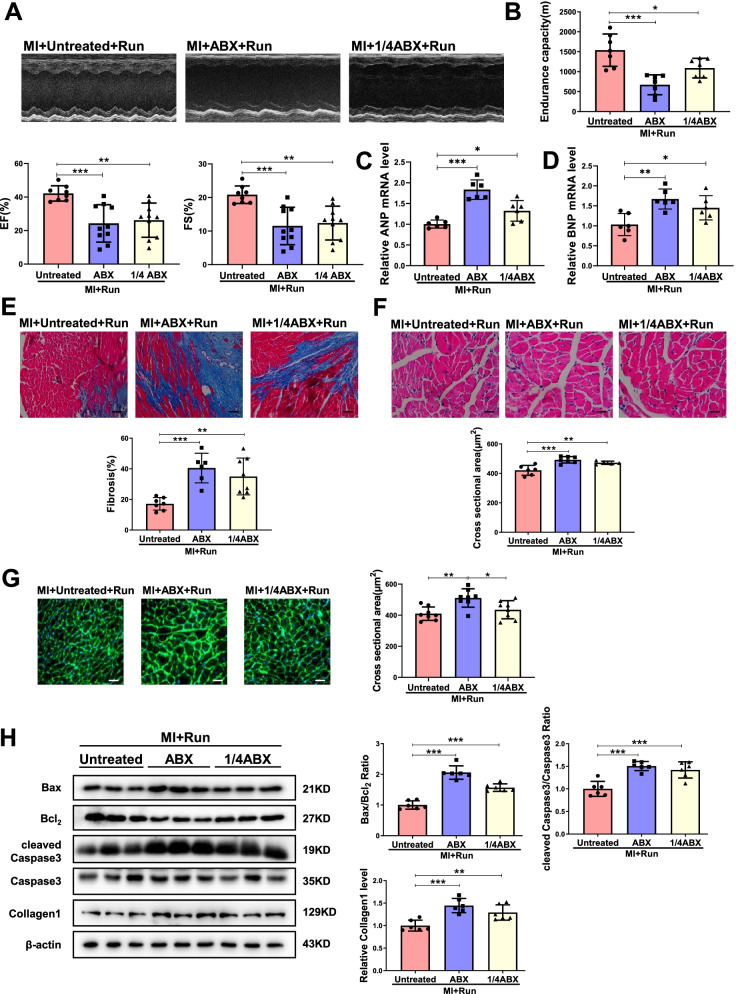
Gut microbiota pre-depletion by antibiotics abolishes the protection of running in MI. **A** Antibiotics (ABX and 1/4 ABX) decreased EF and FS in MI + Run mice (*n* = 8:10:10). **B** Antibiotics decreased endurance capacity in MI + Run mice (*n* = 7:7:7). **C**, **D** Antibiotics increased cardiac ANP and BNP mRNA expression in MI + Run mice (*n* = 6:6:6). **E** Antibiotics increased cardiac fibrosis in MI + Run mice (*n* = 7:6:8). **F** Antibiotics increased cardiac cross sectional area in MI + Run mice by H&E (*n* = 6:7:5). **G** Antibiotics increased cardiac cross sectional area in MI + Run mice by WGA (*n* = 8:8:8). **H** Antibiotics increased Bax/Bcl_2_, cleaved Caspase3/Caspase3 and Collagen1 in MI + Run mice (*n* = 6:6:6). Scale bar: 50 μm in E and 25 μm in F and G. Data were represented as mean ± SD. Significant differences were assessed by one-way ANOVA followed by Bonferroni's multiple comparisons test. *p < 0.05, **p < 0.01, ***p < 0.001

To investigate if gut microbiota in general is responsible for the beneficial effects of exercise training in cardiac dysfunction post-MI, we administered oral antibiotics to male mice 1-week post-MI for 7 days to pre-deplete the gut microbiota and started exercise training for another 8 weeks (Fig. S5A). As the maximal dose give to the mice, which was named ABX mice (ABX treated for 1 week as indicated in Fig. S5A), led to 42% mortality in MI mice, we also used a quarter of this dose (1/4 ABX group, 1/4 ABX treated for 9 weeks as indicated in Fig. S5A) that did not result in post-MI deaths (Fig. S5B and C). As indicated in Fig. 3A, both ABX administration and 1/4 ABX administration impaired cardiac function of exercise MI mice, which is evidenced by the loss of beneficial effects of exercise training, including EF and FS (Fig. 3A), endurance capacity (Fig. 3B), the mRNA expression levels of ANP and BNP (Fig. 3C and D), cardiac fibrosis, cross-sectional cardiomyocyte area, and apoptosis (Fig. 3E–H). Of note, ABX or 1/4 ABX had no significant effect on cardiac function in MI mice (Fig. S6). Thus, these data confirm that gut microbiota are involved in the protective effects of exercise training in cardiac dysfunction post-MI.

We also further confirmed the effects of ABX or 1/4 ABX in gut microbiota in MI by Pan analysis and alpha diversity, which showed that as compared to MI groups, ABX or 1/4 ABX decreased levels of gene enrichment (Fig. S7A and B, Additional file 1: Table S8-S11). Based on the Community barplot analysis, the relative abundance of gut microbiota at the phylum level was changed by ABX or 1/4 ABX (Fig. S7C and D, Additional file 1: Table S12–13). Moreover, the changes of genus ABX or 1/4 ABX in MI followed by running training were also showed in Fig. S8 and Additional file 1: Table S14.

### Fecal microbial transplantation from exercise training post-MI is a transmissible trait

**Fig. 4:**
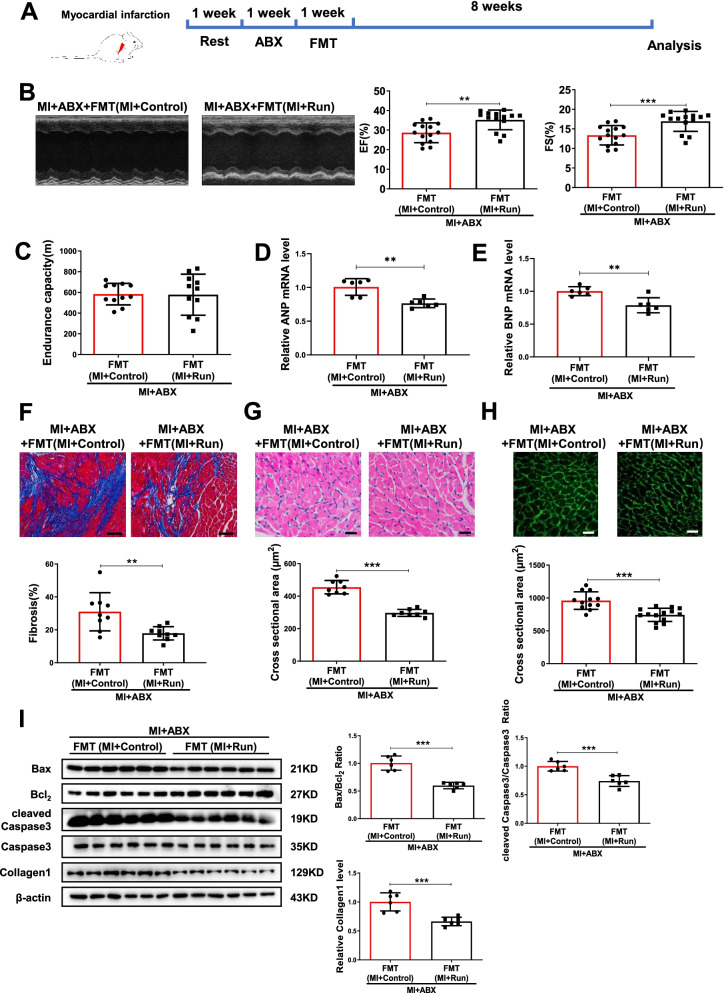
FMT recovers the protection of running in MI. **A** The schedule of FMT after ABX treated. **B** FMT from MI + Run increased EF and FS compared to FMT from MI + Control post-MI (*n* = 14:14). **C** FMT from MI + Run increased endurance capacity compared to FMT from MI + Control post-MI (*n* = 11:11). **D**, **E** FMT from MI + Run decreased cardiac ANP and BNP mRNA expression compared to FMT from MI + control post-MI (*n* = 6:6). **F** FMT from MI + Run decreased cardiac fibrosis compared to FMT from MI + control post-MI (*n* = 9:9). **G** FMT from MI + Run decreased cardiac cross sectional area compared to FMT from MI + control post-MI by H&E (*n* = 8:8). **H** FMT from MI + Run decreased cardiac cross sectional area compared to FMT from MI + control post-MI by WGA (*n* = 12:14). **I** FMT from MI + Run decreased Bax/Bcl_2_, cleaved Caspase3/Caspase3 and collagen1 compared to FMT from MI + Control post-MI (*n* = 6:6). Scale bar: 50 μm in F and 25 μm in **G** and **H**. Data were represented as mean ± SD. Significant differences were assessed by two-tailed student *t* test. **p < 0.01, ***p < 0.001

Many diseases’ severities have been previously reported to be influenced by microbiota transplantation such as hypertension, metabolic syndrome, and MI [6, 15, 28]. Here, we sought to determine whether the beneficial effect of exercise training in MI is transmissible with fecal microbial transplantation by using fecal samples from exercised MI mice (Fig. 4A). Using echocardiographic analysis, we observed that mice receiving microbiota transplants from exercised-MI mice had better cardiac function as compared to those mice receiving microbiota transplants from MI mice (Fig. 4B). Although no significant difference of endurance capacity was observed, mice receiving microbiota transplants from exercised-MI mice had attenuated cardiac hypertrophy and fibrosis, decreased cross-sectional cardiomyocyte area, and apoptosis (Fig. 4C–I).

We further investigated the effects of FMT in community richness and structure in MI mice treated with ABX. Pan analysis showed that mice received microbiota transplants from exercised-MI mice or non-exercised-MI mice could both increase community richness (Fig. S9A). Based on the community barplot analysis, the relative abundance of gut microbiota at the phylum level and genus level was changed by FMT (Fig. S9B and C, Additional file 1: Table S15–16). We also used PCoA and NMDS analysis based on the relative abundance of genus to examine the community structures of the gut microbiotas, and found that the gut microbiota of mice received microbiota transplants from exercised-MI mice were separated from those mice received microbiota transplants from non-exercised-MI mice (Fig. S9D and E). Moreover, the changes of genus by FMT were also showed in Fig. S10.

### Metabolomics in exercise after MI

We performed MicrobioMET (Metabo-Profile, Shanghai, P.R. China) to analyze metabolomics in mice fecal samples from exercise post-MI. Overall, 116 metabolites including amino acids, fatty acids, indoles, organic acids, and phenols were quantitated using ultraperformance liquid chromatography coupled to tandem mass spectrometry (UPLC-MS/MS) system (Additional file 1: Table S17).

**Fig. 5:**
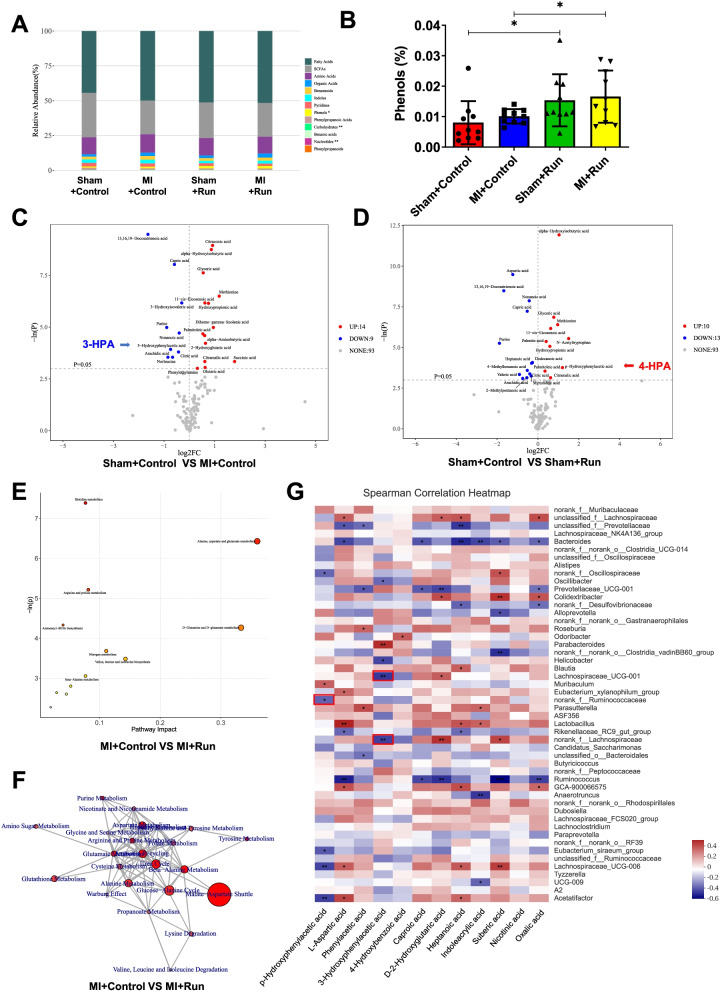
3-HPA and 4-HPA are identified to be involved in the protection of running in MI. OPLS-DA Model Discrimination based on metabolic profiles in fecal samples. **A** The relative abundance of each metabolite classes in different groups (*n* = 10:10:10:10). **B** The abundance of phenols in different groups. **C** Volcano plot of univariate statistics across Sham + control group and MI + control group. **D** Volcano plot of univariate statistics across Sham + control group and Sham + Run group. **E** Pathway analysis bubble plot by mmu set in MI + Control vs MI + Run. **F** Network for statistically significant changed pathways in MI + control vs. MI + Run. **G** The relationship between metabolites and the top 50 genera in four groups was estimated by Spearman’s correlation analysis. Those with low correlated (|*r*|< 0.1) were not shown. Genera and cardiac function index were distinguished as positive (red) and negative (blue) correlation. *p < 0.05; **p < 0.01; ***p < 0.001

Metabolites may have similar functions because they are of the same nature. We divided 116 metabolites into 13 categories according to their nature. Relative abundance of metabolites category showed that phenols were increased in the MI + Run group compared to MI group (Fig. 5A and B, Additional file 1: Table S18). 3-Hydroxyphenylacetic acid (3-HPA) and p-Hydroxyphenylacetic acid (4-HPA) are the main component of phenols. 3-HPA contents were decreased in MI group as compared to control group, and 4-HPA contents were increased in Sham + Run group compared to control group (Fig. 5C and D, Additional file 1: Table S19 and S20). Consistently, 3-HPA was decreased in MI + control group as compared to Sham + control group, and 4-HPA was increased in MI + Run group as compared to MI + control group by volcano plot of OPLS-DA model (Fig. S11). These findings suggested 3-HPA and 4-HPA mediate beneficial effects of microbiota transplants from exercised MI mice.

To discriminate the metabolic profiles across groups, we performed clustering analyses based on orthogonal partial least square discriminant analysis (OPLS-DA). OPLS-DA was a partitioning of the X-data facilitates model interpretation and model prediction. The fecal samples from distinct groups were largely separated according to the OPLS-DA plots. Forty-two significant variable metabolites were identified between Sham + control group and MI + control group, while 37 significant variable metabolites were found between MI + control group and MI + Run group (Fig. S12, Additional file 1: Table S21, S22). Furthermore, we identified 4_Hydroxybenzoic acid (4-HBA) as a significant variable metabolite in both Sham + control and MI + Run group as compared to MI + control group (Additional file 1: Table S21, S22). These findings suggested 4-HBA might play a role in mediating beneficial effects of microbiota transplants from exercised MI mice.

To identify metabolic pathways that potentially play a role in the protective effects of exercise post-MI, we analyzed the pathway enrichment in both Sham + control and MI + Run group as compared to MI + control group, as well as Sham + Run group compared to Sham + control group. Interestingly, Alanine, aspartate, and glutamate metabolism pathway had a significant impact on MI + control vs. MI + Run (Fig. 5E and F, Fig. S13, Additional file 1: Table S23-S28).

We examined the relationship between 12 representative metabolites and the gut microbial using correlation analysis. There was 11 metabolomics significantly correlated with 31 genera in all samples. Among them, 4-HPA, 3-HPA, and 4-HBA were significantly correlated with 6 genera, 5 genera, and 1 genera, respectively (Fig. 5G). Interestingly, 11 metabolomics was found to be significantly correlated with 7 genera which were significant changed in MI + Run group compared to MI + control group, including *Lachnospiraceae*, *Bacteroides*, *Roseburia*, *Lachnospiraceae_UCG-001*, *Ruminococcaceae*, *g_norank_Lachnospiraceae* and *Ruminococcus* (Additional file 1: Table S29). Among them, 3-HPA was negatively correlated with *Lachnospiraceae_UCG-001* and *Lachnospiraceae*, while 4-HPA was negatively linked to *Ruminococcaceae*.

Collectively, these findings suggest that 3-HPA, 4-HPA, and 4-HBA may potentially contribute to the protective effects of exercise post-MI.

### Two metabolites, 3-HPA and 4-HBA, protect cardiac dysfunction through activating NRF2

**Fig. 6:**
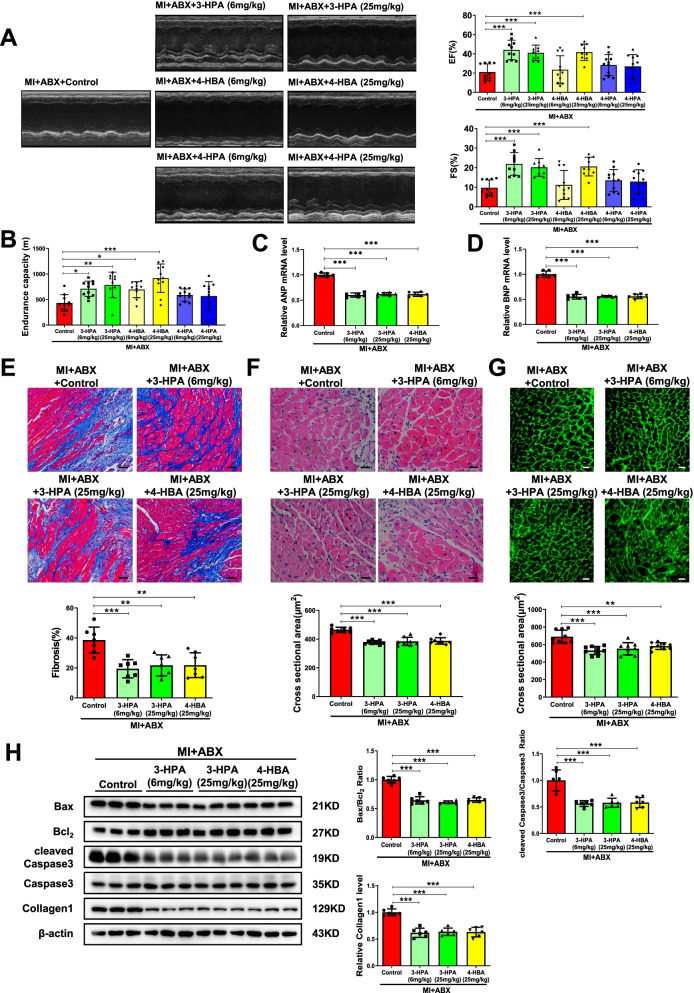
3-HPA and 4-HBA protect against cardiac dysfunction after MI. **A** 3-HPA and 4-HBA increased EF and FS post-MI (*n* = 10:10:9:11:9:11:11). **B** 3-HPA and 4-HBA increased endurance capacity post-MI (n = 10:11:10:10:11:11:11). **C**, **D** 3-HPA and 4-HBA decreased cardiac ANP and BNP mRNA expression post-MI (*n* = 6:6:6:6). **E** 3-HPA and 4-HBA decreased cardiac fibrosis post-MI (*n* = 7:7:7:7). **F** 3-HPA and 4-HBA decreased cardiac cross sectional area post-MI by H&E (*n* = 8:8:8:8). **G** 3-HPA and 4-HBA decreased cardiac cross sectional area post-MI by WGA (*n* = 9:9:9:9). **H** 3-HPA and 4-HBA decreased Bax/Bcl_2_, cleaved Caspase3/Caspase3 and Collagen1 post-MI (*n* = 6:6:6:6). Scale bar: 50 μm in E and 25 μm in F and G. Data were represented as mean ± SD. Significant differences were assessed by one-way ANOVA followed by Bonferroni's multiple comparisons test. *p < 0.05, **p < 0.01, ***p < 0.001

As 3-HPA, 4-HBA, and 4-HPA were indicated to potentially play a role in mediating beneficial effects of microbiota transplants from exercised MI mice, we checked whether their positive effects in cardiac function post-MI could be directly transferred. Every metabolite was tested at two doses, 6 mg/kg and 25 mg/kg. 3-HPA at both doses and 4-HBA at a dose of 25 mg/kg could significantly lead to improved EF and FS post-MI while 4-HPA failed to have beneficial effects (Fig. 6A). Besides, 3-HPA and 4-HBA at both doses could enhance endurance capacity in post-MI mice (Fig. 6B). Moreover, 3-HPA at both doses and 4-HBA at a dose of 25 mg/kg significantly attenuated cardiac hypertrophy and fibrosis, decreased cross-sectional myocardium area, and apoptosis (Fig. 6C–H). Therefore, supplementing these two metabolites, 3-HPA and 4-HBA, protects cardiac dysfunction post-MI.

**Fig. 7:**
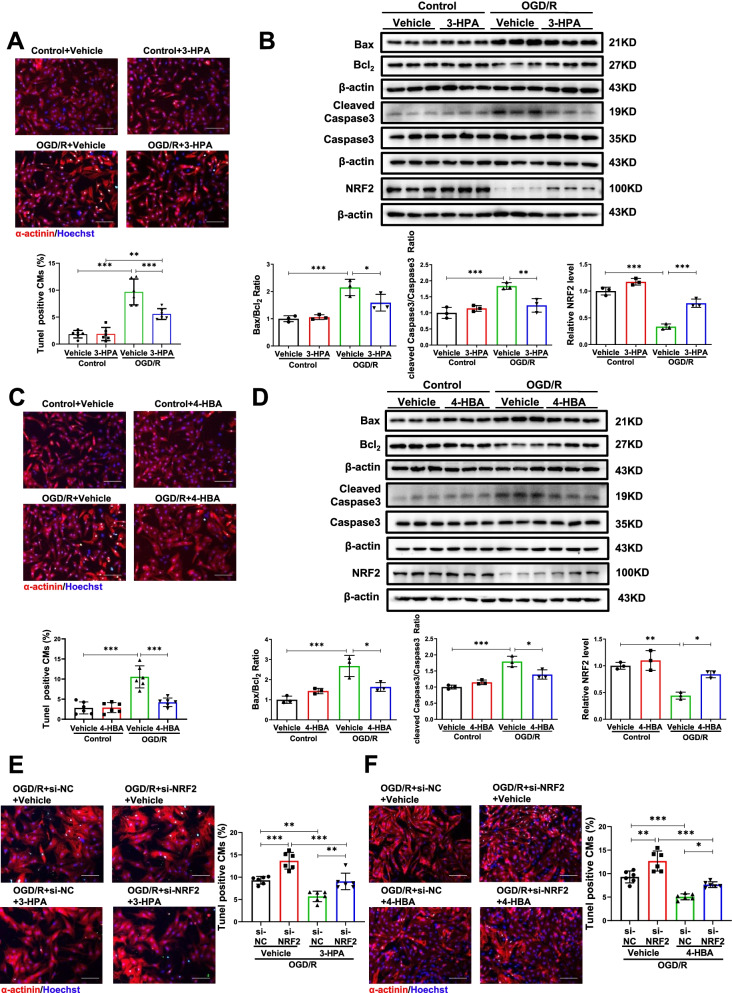
3-HPA and 4-HBA mediate cardiac protection through activating NRF2. **A**, **B** 3-HPA (100 μM, 24 h) reduced the percentage of TUNEL staining positive cardiomyocytes (*n* = 6:6:6:6), Bax/Bcl_2_, cleaved Caspase3/Caspase3, NRF2 (*n* = 3:3:3:3) in NRCMs under oxygen glucose deprivation/reperfusion (OGD/R). **C**, **D** 4-HBA (100 μM, 24 h) decreased the percentage of TUNEL staining positive cardiomyocytes (*n* = 6:6:6:6), Bax/Bcl_2_, cleaved Caspase3/Caspase3, NRF2 (*n* = 3:3:3:3) in NRCMs under OGD/R. **E**, **F** Inhibition of NRF2 increased the percentage of TUNEL staining positive cardiomyocytes decreased by 3-HPA and 4-HBA in NRCMs under OGD/R (*n* = 6:6:6:6). Scale bar: 100 μm. Data were represented as mean ± SD. Significant differences were assessed by two-way ANOVA followed by Bonferroni's multiple comparisons test. *p < 0.05, **p < 0.01, ***p < 0.001 versus respective control

We further checked the effect of 3-HPA and 4-HBA in vitro. As expected, 3-HPA and 4-HBA decreased NRCMs apoptosis induced by OGD/R as indicated by a reduction in TUNEL staining positive cardiomyocytes and the ratios of Bax/Bcl2 and cleaved Caspase3/Caspase3 (Fig. S14 and Fig. 7A–D). As NRF2 has been reported to be a target of phenols [29], and NRF2 could protect against MI and apoptosis induced by OGD/R [30, 31], we further analyzed whether NRF2 mediated the protective effects of 3-HPA and 4-HBA on apoptosis. 3-HPA and 4-HBA increased the expression of NRF2, and inhibition of NRF2 prevented the protective effects of 3-HPA and 4-HBA on apoptosis (Fig. 7B, D–F). Together, our findings illustrate that 3-HPA and 4-HBA protect cardiomyocytes apoptosis by activating NRF2.

## Discussion

There is a growing recognition of the contribution of gut microbiota to health and disease susceptibility. Herein, we described exercise training improved cardiac dysfunction and rescued gut microbial richness and community structure post-MI. Besides, we observed that two metabolites, 3-HPA and 4-HBA, played potential roles in mediating beneficial effects of exercise in MI mice through NRF2.

Potential microbiota-altering therapies can lead to new approaches for preventing and managing cardiovascular diseases [32, 33]. The gut microbiome is primarily composed of species within the Bacteroidetes, Firmicutes, Actinobacteria, Proteobacteria, and Cerrucomicrobia phylae [34]. Furthermore, Archaea, Verrucomicrobia, and Spirochaetes were reported in global gut metagenomes [35]. In the total bacterial species, the most abundant species are Bacteroidetes and Firmicutes [36]. Microbiota richness and diversity are important to maintain stability and performance and new biomarkers of health [37]. Loss of gut flora richness and biodiversity are associated with various diseases [3, 38]. For example, hypertension patients dramatically decreased microbial richness and diversity [39]. In heart failure patients, a significantly decreased diversity of the intestinal microbiome has been observed [40]. Similarly, we found that MI decreased community richness of gut microbial based on Pan analysis. On genus level, Lactobacillus, Alistipes, and Turicibacter are more abundant in mouse gut microbiota [41]. In our study, we found that *Lachnospiraceae_UCG-001* was increased in MI while *Alistipes*, *Ruminococcus*, *Allobaculum*, and *Oscillospiraceae UCG-005* were decreased in MI. *Lachnospiraceae* has been reported to be higher in 7-day post-MI in rat, which is consistent with our data [42]. *Allobaculum*, an important healthy component of the mouse microbiome, was found to be decreased in obese mice or mice that lost circadian rhythm [43, 44]. Here, we found that *Allobaculum* was decreased in MI and could be increased by running training in MI mice, which is consistent with the report that *Allobaculum* could be increased with exercise [45]. On the whole, our findings on gut microbiota in MI are supported by many hints in previous researches.

Physical exercise has been demonstrated to be able to modulate gut microbiota and increase the abundance of beneficial microbial species [46–48]. The microbiome of professional athletes exhibits higher richness compared to sedentary controls [49]. Consistently, we found that running treatments could increase community richness of gut microbial. In coronary heart disease, Phyla Bacteroidetes and Proteobacteria were decreased, whereas the Phyla Firmicutes were increased [50]. Interestingly, we found that running treatments could increase Bacteroidetes and reduce Firmicutes in MI + RNU group compared with MI group. Furthermore, we discovered that gut microbiota was responsible for the beneficial effects of exercise training in cardiac dysfunction post-MI, since orally antibiotics could prevent the beneficial effects of exercise training in cardiac fibrosis, cross-sectional area, and apoptosis. In human AMI patients, a higher microbial richness and diversity was found in the systemic microbiome of ST-segment elevation MI patients’ blood [51]. However, our work illustrated the richness of gut microbiota was decreased 8-week post-MI but was increased by running training in MI. In addition, MI and running did not change the community diversity of gut microbial. We speculate that the content of intestinal flora is distinct in different stages of MI. Among Bacteroidetes, *Alloprevotella* was associated with decreased lifetime CVD risk. Among Firmicutes,* Tyzzerella *was enriched among those with high CVD risk profile [52, 53]. But we expound no association between *Alloprevotella*, *Tyzzerella_3*, and cardiac function. MI was associated with a reorganization of the gut microbial community, such as a reduction in *Lactobacillus*. Supplementation of different species of *Lactobacillus* in rats showed cardioprotective effects when administered after MI [15]. In contrast, we found no association between *Lactobacillus* and cardiac function. Candida was correlated with heart failure severity [54]. But we did not observe this difference of Candida in MI mice. In general, our findings on the relationships between exercise, gut microbiota, and heart are consistent or inconsistent with previous studies. Further researches are needed to confirm the deeply link.

Gut microbiome functions like an endocrine organ, generating bioactive metabolites such as short-chain fatty acids and bile acids that can impact the host physiology [55]. Diverse roles of the gut microbiota in human health and disease have been recognized [56]. Mounting evidence in mice and human is accumulating showing that gut microbiota is linked with cardiovascular health [57, 58]. Metabolic therapy could confer benefit for treating heart failure [59]. Microbial transplantation studies could provide strong evidence to support the contribution of gut microbiota in host physiological processes and disease risks [60, 61]. Metabolites play important roles in individual physiological stress, disease process, and drug development. The application of metabolites in cardiovascular disease is a rapidly expanding field [62]. Metabolite profiling in Finland, Southall, and Brent, British populations identified phenylalanine, monounsaturated fatty acids, and polyunsaturated fatty acids as biomarkers for cardiovascular risk [63]. Glycerophosphocholine metabolites modulate atherosclerosis and thus risk for cardiovascular disease [64]. Our work analyzed metabolomics in mice fecal samples from exercise after MI and screened 12 variable significant metabolites including 3-HPA and 4-HBA. 3-HPA is reported as a strong predictor of autism spectrum disorders [65]. Oral administration of 4-HBA decreased blood glucose levels in the diabetic rat by increasing glucose consumption [66]. Exercise induced alterations in metabolites such as alpha-tocopherol, myocardial high-energy phosphate metabolites in patients with Chagas heart disease [67, 68]. However, the role of 3-HPA and 4-HBA in exercise and MI remains unknown. Herein, we discovered two metabolites, 3-HPA and 4-HBA, that can protect for cardiac dysfunction post-MI. In NRCMs, 3-HPA and 4-HBA could decrease apoptosis induced by OGD/R through activating NRF2. Further investigations are needed to find out the role of NRF2 in exercised MI mice.

In conclusion, our study revealed that gut microbiota mediated the protective effects of exercise after myocardial infarction based on the 16S rRNA sequencing analysis, oral antibiotics administration, and fecal microbial transplantation. We identified two metabolites from the fecal of exercised mice post-MI, 3-HPA and 4-HBA, protecting against cardiac dysfunction post-MI and apoptosis through NRF2. Our study provides insights into the composition of the gut microbiota in exercise and MI mice, enhances our understanding of the effect of 3-HPA and 4-HBA on MI, and launch approaches to modulate cardiovascular diseases by exercise, microbiome, and metabolites.

## Conclusion

Together, our study provides new insights into the role of gut microbiome in exercise protection to MI, offers opportunities to modulate cardiovascular diseases by exercise, microbiome, and gut microbiota-derived 3-HPA and 4-HBA.

## Supplementary Information

**Additional file 1: Table S1.** Data production of 49 samples in Sham+Control, MI+Control, Sham+Run, and MI+Run. **Table S2.** Alpha Diversity index among Sham+Control, MI+Control, Sham+Run, and MI+Run. **Table S3.** The difference of Alpha Diversity index among Sham+Control, MI+Control, Sham+Run, and MI+Run. **Table S4.** Relative abundance profile at the phylum level among Sham+Control, MI+Control, Sham+Run, and MI+Run. **Table S5.** Relative abundance profile at the genus level among Sham+Control, MI+Control, Sham+Run, and MI+Run. **Table S6.** Detailed information of genera differently enriched across Sham+Control and MI+Control. **Table S7.** Detailed information of genera differently enriched across MI+Control and MI+Run. **Table S8.** Alpha Diversity index among MI+ABX, MI+ABX+Control, MI+1/4ABX+Control and MI+Untreated+Control. **Table S9.** The difference of Alpha Diversity index among MI+ABX, MI+ABX+Control, MI+1/4ABX+Control and MI+Untreated+Control. **Table S10.** Alpha Diversity index among MI+ABX+Run, MI+1/4ABX+Run and MI+Untreated+Run. **Table S11.** The difference of Alpha Diversity index among MI+ABX+Run, MI+1/4ABX+Run and MI+Untreated+Run. **Table S12.** Relative abundance profile at the phylum level among MI+ABX, MI+ABX+Control, MI+1/4ABX+Control and MI+Untreated+Control. **Table S13.** Relative abundance profile at the phylum level among MI+ABX+Run, MI+1/4ABX+Run and MI+Untreated+Run. **Table S14.** Detailed information of genera differently enriched across MI+ABX+Run, MI+1/4ABX+Run and MI+Untreated+Run. **Table S15.** Relative abundance profile at the phylum level among MI+ABX+PBS, MI+ABX+FMT(MI+Control) and MI+ABX+FMT(MI+Run). **Table S16.** Relative abundance profile at the genus level among MI+ABX+PBS, MI+ABX+FMT(MI+Control) and MI+ABX+FMT(MI+Run). **Table S17.** Quantitative data of 116 metabolites in Sham+Control, MI+Control, Sham+Run, and MI+Run. **Table S18.** The relative abundance of each metabolite classes in Sham+Control, MI+Control, Sham+Run, and MI+Run. **Table S19.** Detailed information of Univariate Statistics across Sham+Control and MI+Control. **Table S20.**.Detailed information of Univariate Statistics across Sham+Control and Sham+RUN. **Table S21.** Detailed information of 42 metabolites differently enriched across Sham+Control and MI+Control. **Table S22.** Detailed information of 37 metabolites differently enriched across MI+Control and MI+Run. **Table S23.** Detailed information of metabolites pathways analysis across MI+Control and MI+Run. **Table S24.** Detailed information of metabolites pathways on network across MI+Control and MI+Run. **Table S25.** Detailed information of pathways analysis across Sham+Control and MI+Control. **Table S26.** Detailed information of metabolites metabolites pathways on network across Sham+Control and MI+Control. **Table S27.** Detailed information of pathways analysis across Sham+Control and Sham+Run. **Table S28.** Detailed information of metabolites metabolites pathways on network across Sham+Control and Sham+Run. **Table S29.** Detailed information of the relationship between metabolites and the top 50 genera

**Additional file 2: Figure S1.** Relative abundance of gut microbiota in different groups, based on the phylum level. **Figure S2.** Relative abundance of gut microbiota based on the genus level among Sham+Control, MI+Control, Sham+Run, and MI+Run. **Figure S3.** Relative abundance of gut microbiota in different groups, based on the genus level. **Figure S4.** The relationship between the top 50 different genera and cardiac function index. **Figure S5.** MI mice displayed dose-dependent mortality after ABX treated. **Figure S6.** Gut microbiota pre-depletion by antibiotics does not affect cardiac function of MI mice. **Figure S7.** Gut microbiota pre-depletion by ABX decreased community richness and changed structure in mice after MI. **Figure S8.** Top 20 different genus across groups in the feces of mice after MI+Untreated+Run, MI+ABX+Run and MI+1/4ABX+Run. **Figure S9.** Fecal microbiota transplantation (FMT) increased community richness and changed structure in mice after MI+ABX. **Figure S10.** Different genus across groups in the feces of mice after FMT from MI+Control, FMI from MI+Run, or without FMT (PBS). **Figure S11.** 3-HPA and 4-HPA are identified by volcano plot of OPLS-DA model. **Figure S12.** 4-HBA is identified by OPLS-DA Model Discrimination. **Figure S13.** Pathway analysis bubble plot and relevant network. **Figure S14.** 3-HPA and 4-HBA decrease apoptosis with indicated duration and concentration.

## Data Availability

The raw reads were deposited into the NCBI Sequence Read Archive (SRA) database (Accession Number: SRP287461). The data for this study were available by contacting the corresponding author upon reasonable request.
