# Supplementary material for: Gut microbiome mediates the protective effects of exercise after myocardial infarction
Source: Microbiome. 2022 May 31;10:82. doi: 10.1186/s40168-022-01271-6 (PMC9153113; doi:10.1186/s40168-022-01271-6)
Supplement: Supplementary file 3 — Additional file 2: Figure S1. Relative abundance of gut microbiota in different groups, based on the phylum level. Figure S2. Relative abundance of gut microbiota based on the genus level among Sham+Control, MI+Control, Sham+Run, and MI+Run. Figure S3. Relative abundance of gut microbiota in different groups, based on the genus level. Figure S4. The relationship between the top 50 different genera and cardiac function index. Figure S5. MI mice displayed dose-dependent mortality after ABX treated. Figure S6. Gut microbiota pre-depletion by antibiotics does not affect cardiac function of MI mice. Figure S7. Gut microbiota pre-depletion by ABX decreased community richness and changed structure in mice after MI. Figure S8. Top 20 different genus across groups in the feces of mice after MI+Untreated+Run, MI+ABX+Run and MI+1/4ABX+Run. Figure S9. Fecal microbiota transplantation (FMT) increased community richness and changed structure in mice after MI+ABX. Figure S10. Different genus across groups in the feces of mice after FMT from MI+Control, FMI from MI+Run, or without FMT (PBS). Figure S11. 3-HPA and 4-HPA are identified by volcano plot of OPLS-DA model. Figure S12. 4-HBA is identified by OPLS-DA Model Discrimination. Figure S13. Pathway analysis bubble plot and relevant network. Figure S14. 3-HPA and 4-HBA decrease apoptosis with indicated duration and concentration. [file 40168_2022_1271_MOESM2_ESM.docx]

**Supplemental Figure Legends**

**
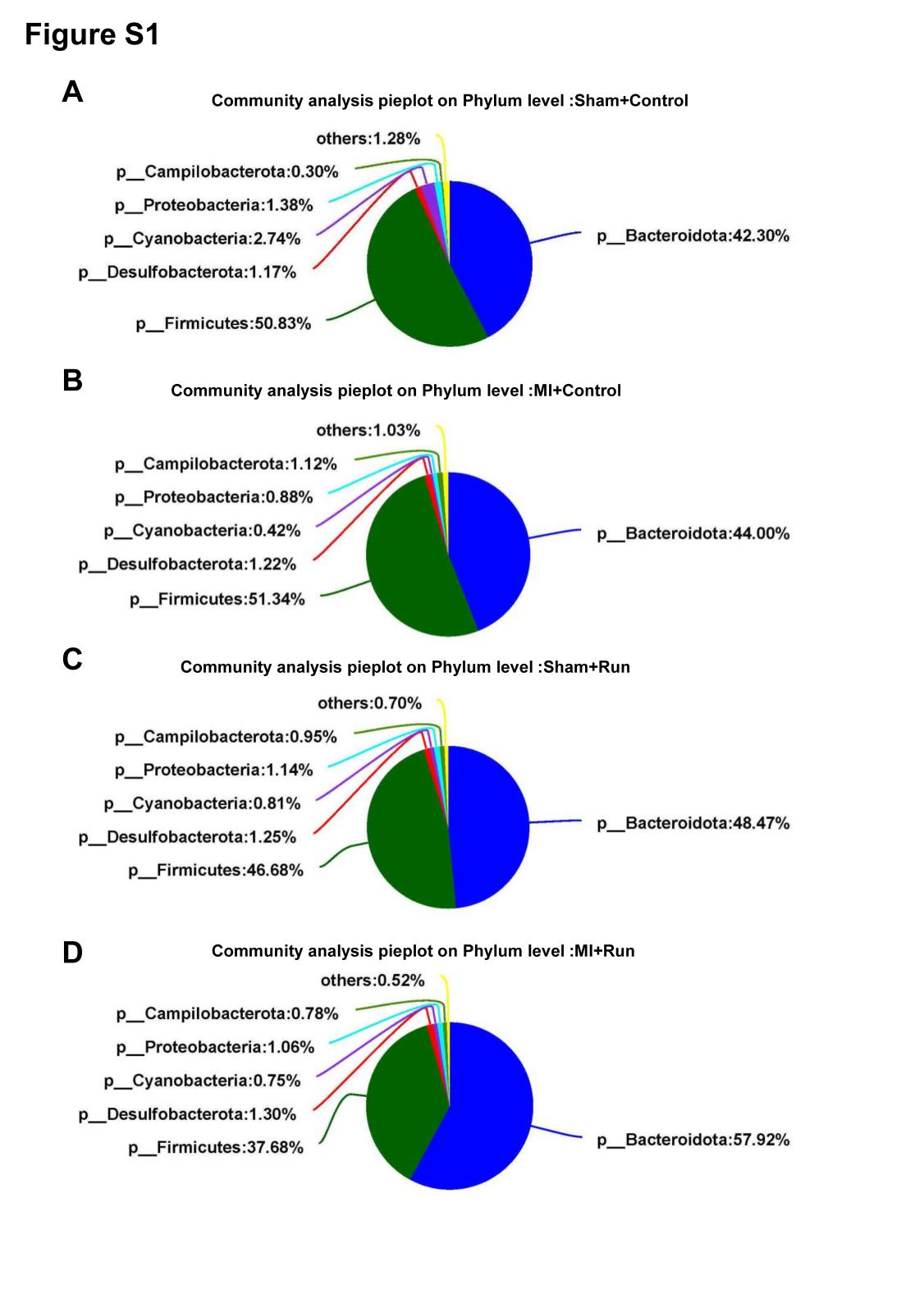
**

**Figure S1. Relative abundance of gut microbiota in different groups, based on the phylum level**

**A**, community analysis pieplot on Phylum level in Sham+Control group. **B**, community analysis pieplot on Phylum level in MI+Control. **C**, community analysis pieplot on Phylum level in Sham+Run. **D**, community analysis pieplot on Phylum level in MI+Run.

**
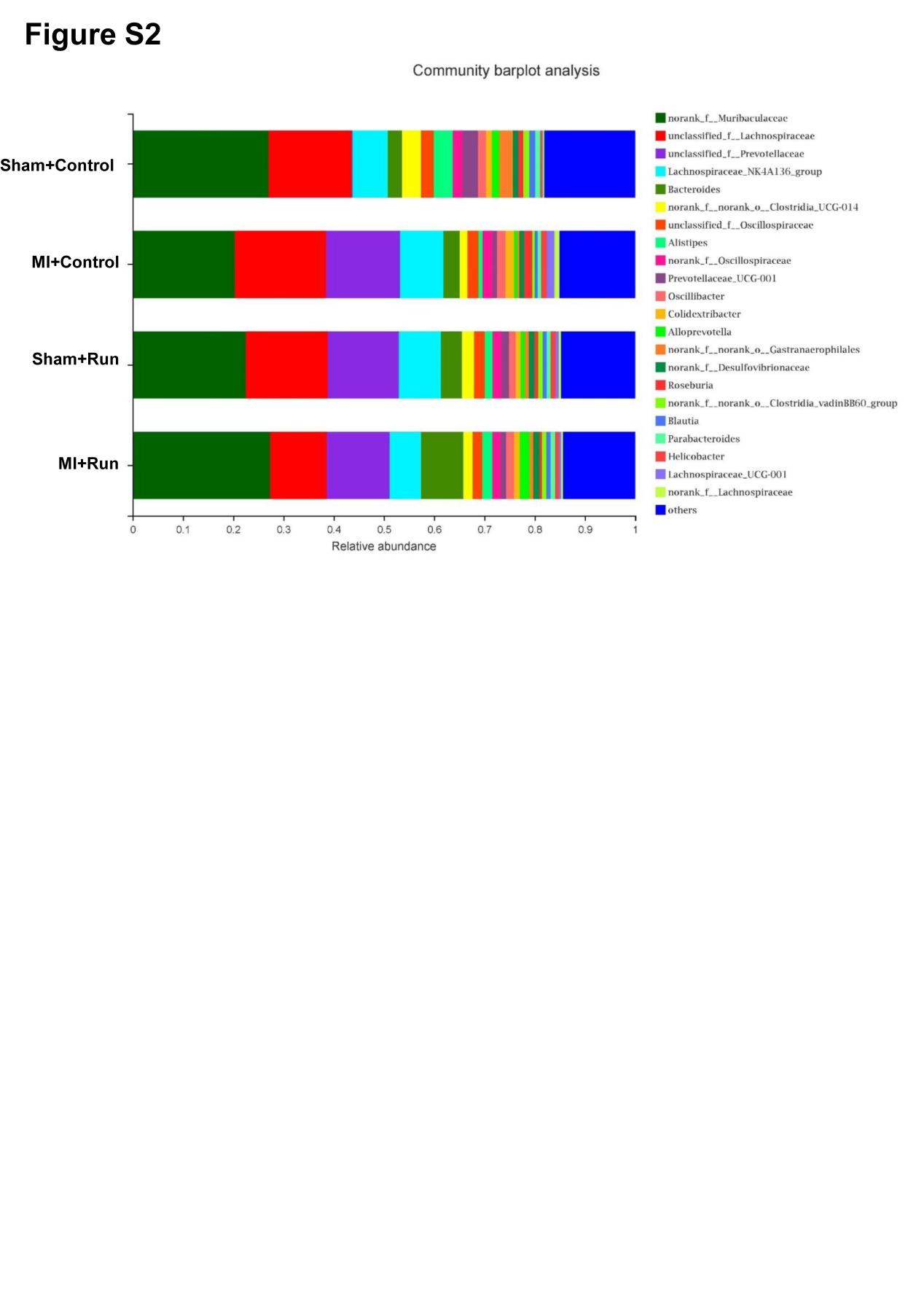
**

**Figure S2. Relative abundance of gut microbiota based on the genus level among Sham+Control, MI+Control, Sham+Run, and MI+Run**

**
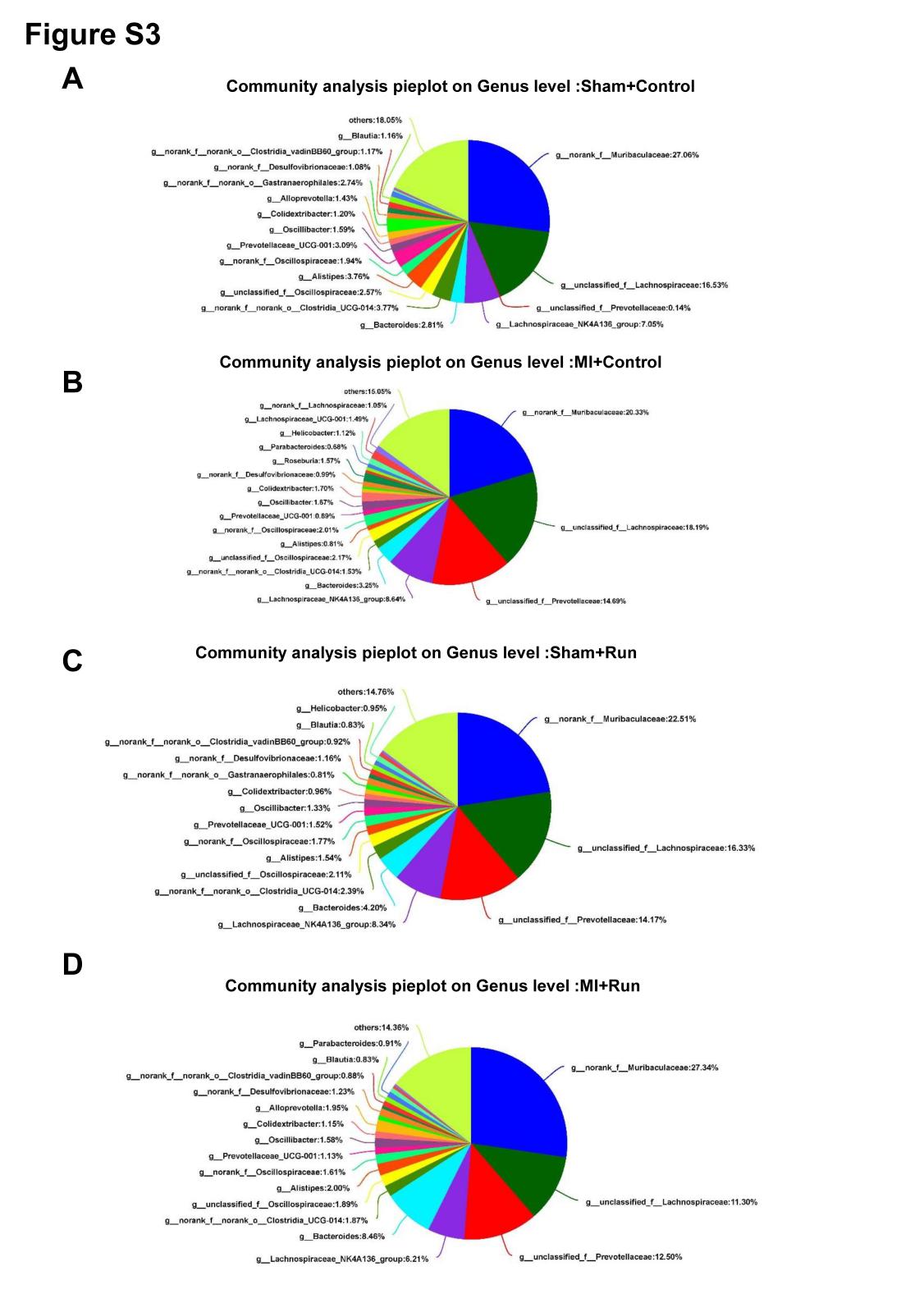
**

**Figure S3. Relative abundance of gut microbiota in different groups, based on the genus level**

**A**, community analysis pieplot on genus level in Sham+Control group. **B**, community analysis pieplot on genus level in MI+Control. **C**, community analysis pieplot on genus level in Sham+Run. **D**, community analysis pieplot on genus level in MI+Run.

**
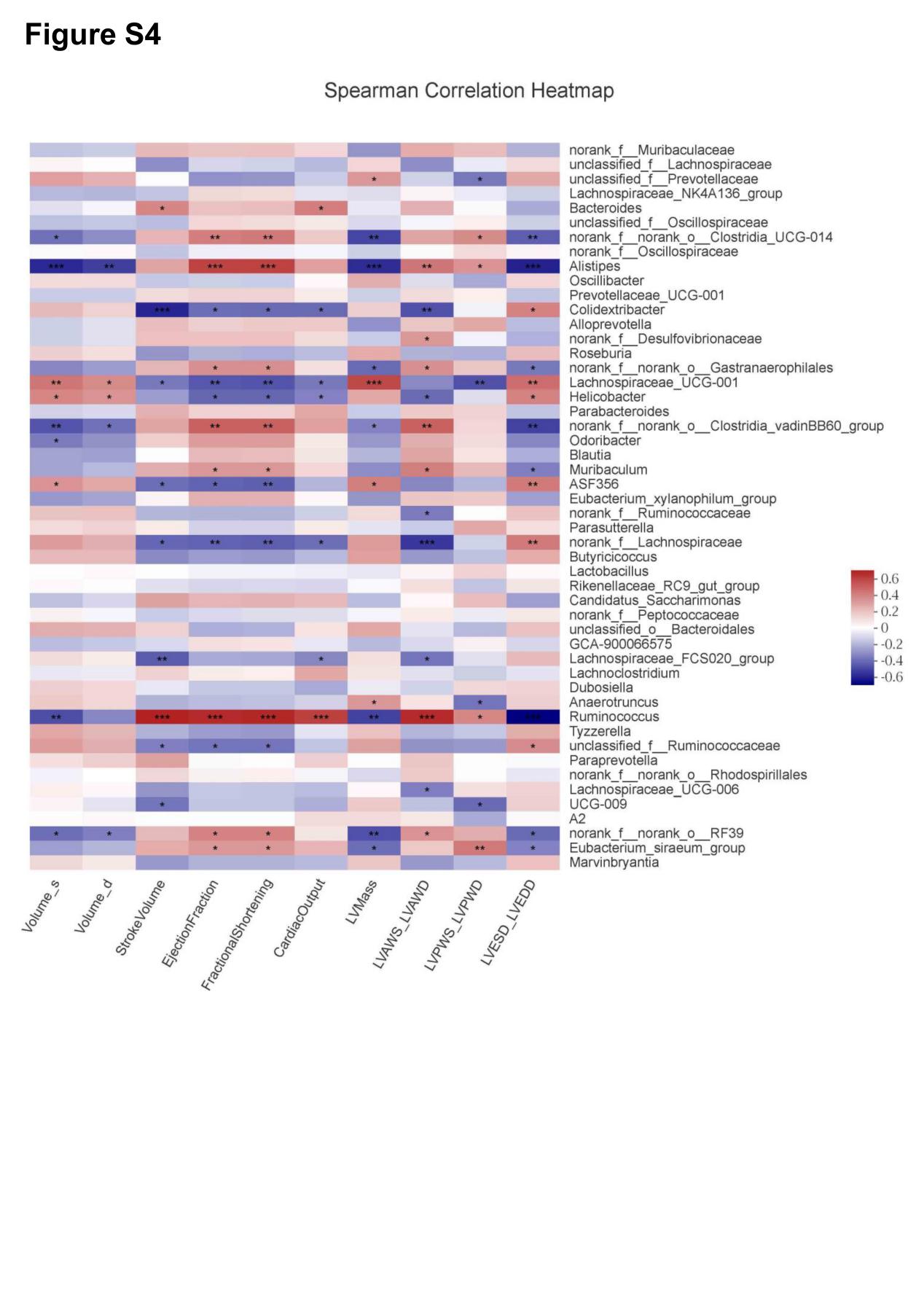
**

**Figure S4. The relationship between the top 50 different genera and cardiac function index.**

The relationship between cardiac function index and the top 50 genera in all samples is estimated by Spearman’s correlation analysis. Cardiac function index filtered by Variance Inflation Factor. Genera and cardiac function index are distinguished as positive (red) and negative (blue) correlation. *, P<0.05; **, P<0.01; ***, P<0.001.


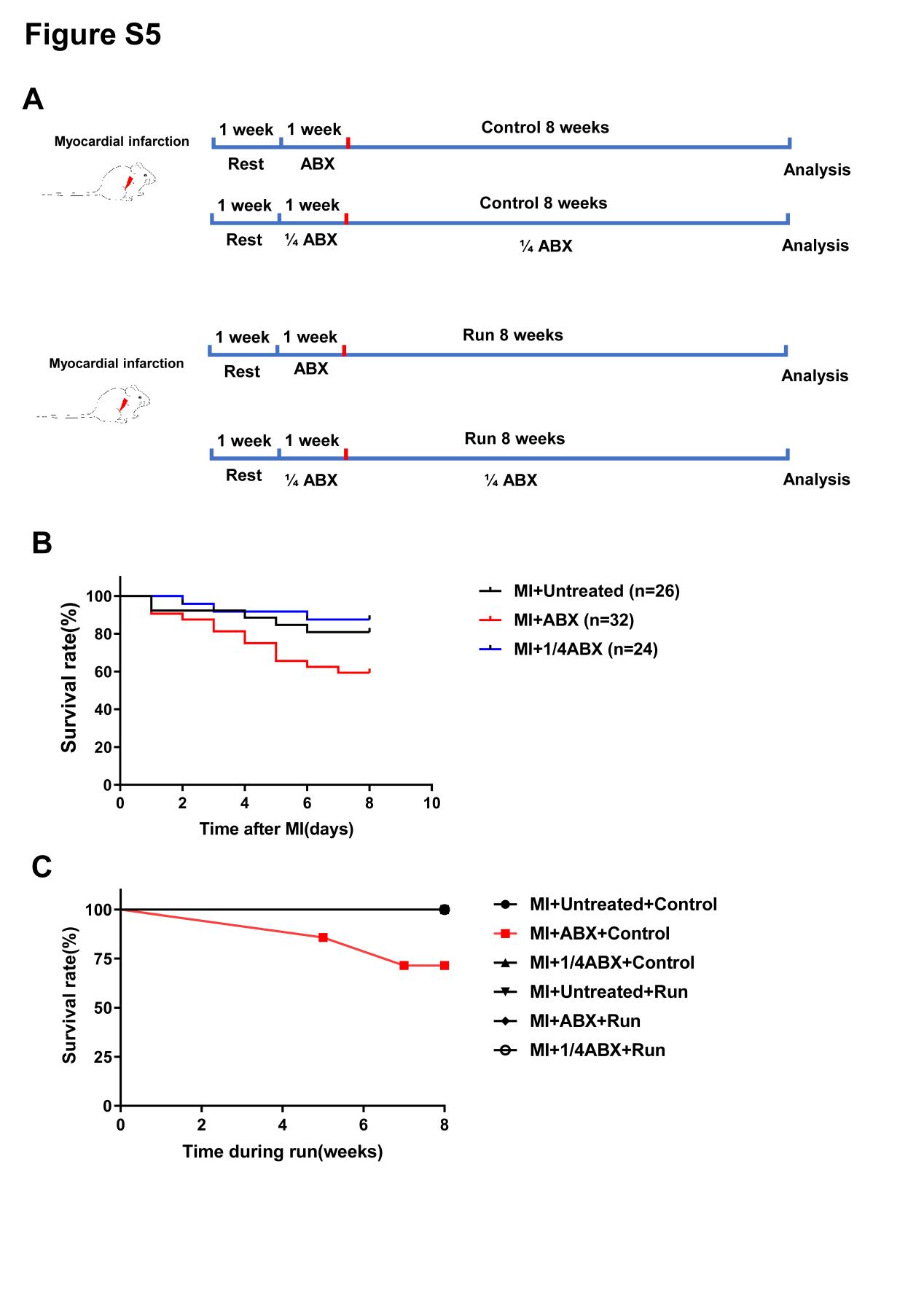


**Figure S5**. **MI mice displayed dose-dependent mortality after ABX treated**

**A**, the schedule of ABX treated and running training after ABX treated. B, the statistics of mortality of ABX mice before running training (n=26:32:24). Mice with similar body weight as selected for experiments in C. C, the statistics of mortality of ABX mice during running training (n=10:9:12:11:11:10).


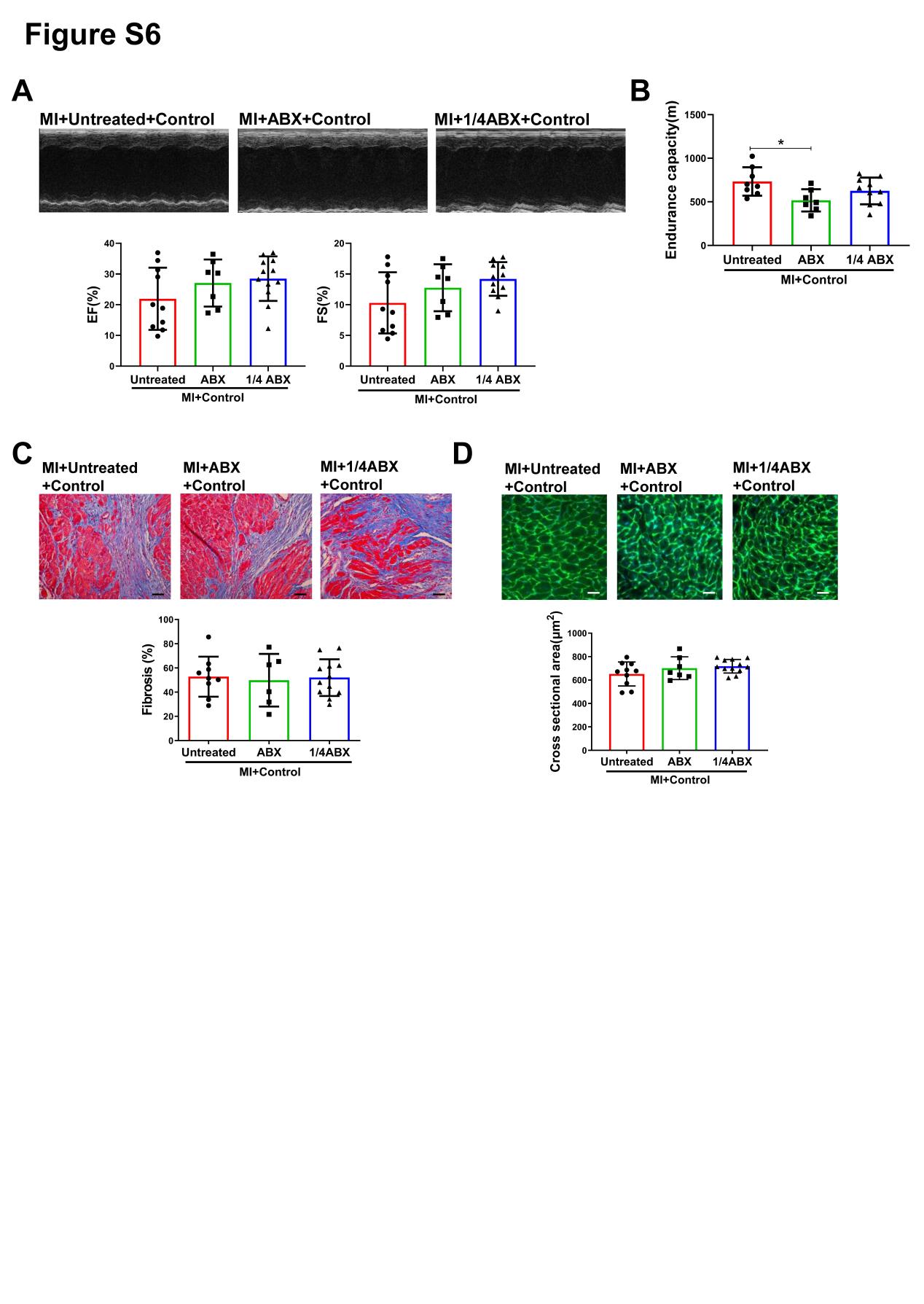


**Figure S6. Gut microbiota pre-depletion by antibiotics does not affect cardiac function of MI mice**

**A**, antibiotics (ABX and 1/4 ABX) do not affect EF and FS in MI+Control mice (n=10:7:12). **B**, ABX decreased while 1/4 ABX do not affect endurance capacity in MI+Control mice (n=8:7:10). **C**, antibiotics do not affect cardiac fibrosis in MI+Control mice (n=9:6:12). **D**, antibiotics do not affect cardiac cross sectional area in MI+Control mice by WGA (n=10:7:12).

Scale bar: 50 μm in C and 25 μm in D. Data were represented as mean ± SD. Significant differences were assessed by one-way ANOVA followed by Bonferroni's multiple comparisons test. *: p<0.05.


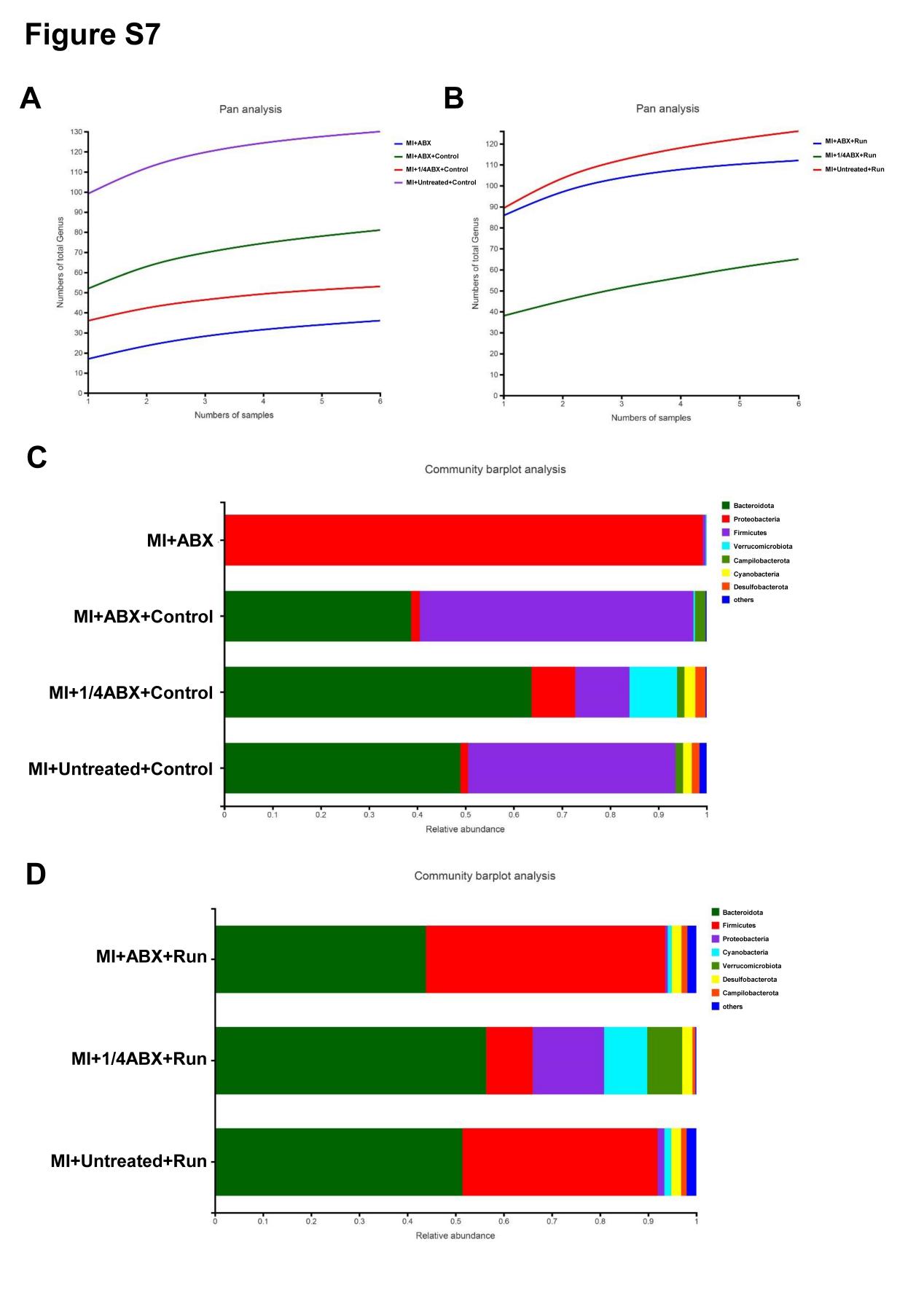


**Figure S7. Gut microbiota pre-depletion by ABX decreased community richness and changed structure in mice after MI**

**A**, Pan analysis for four groups based on genus level (n=6 per group). **B**, Pan analysis for three groups based on genus level (n=6 per group). **C,** fecal bacterial community at the phylum level among four groups. MI+ABX, samples were collected immediately after ABX treatment for 1 week; The other three groups, samples were collected 8 weeks after ABX, 1/4 ABX or Untreated. **D,** fecal bacterial community at the phylum level among three groups. Samples were collected 8 weeks after ABX, 1/4 ABX or Untreated (with running training).


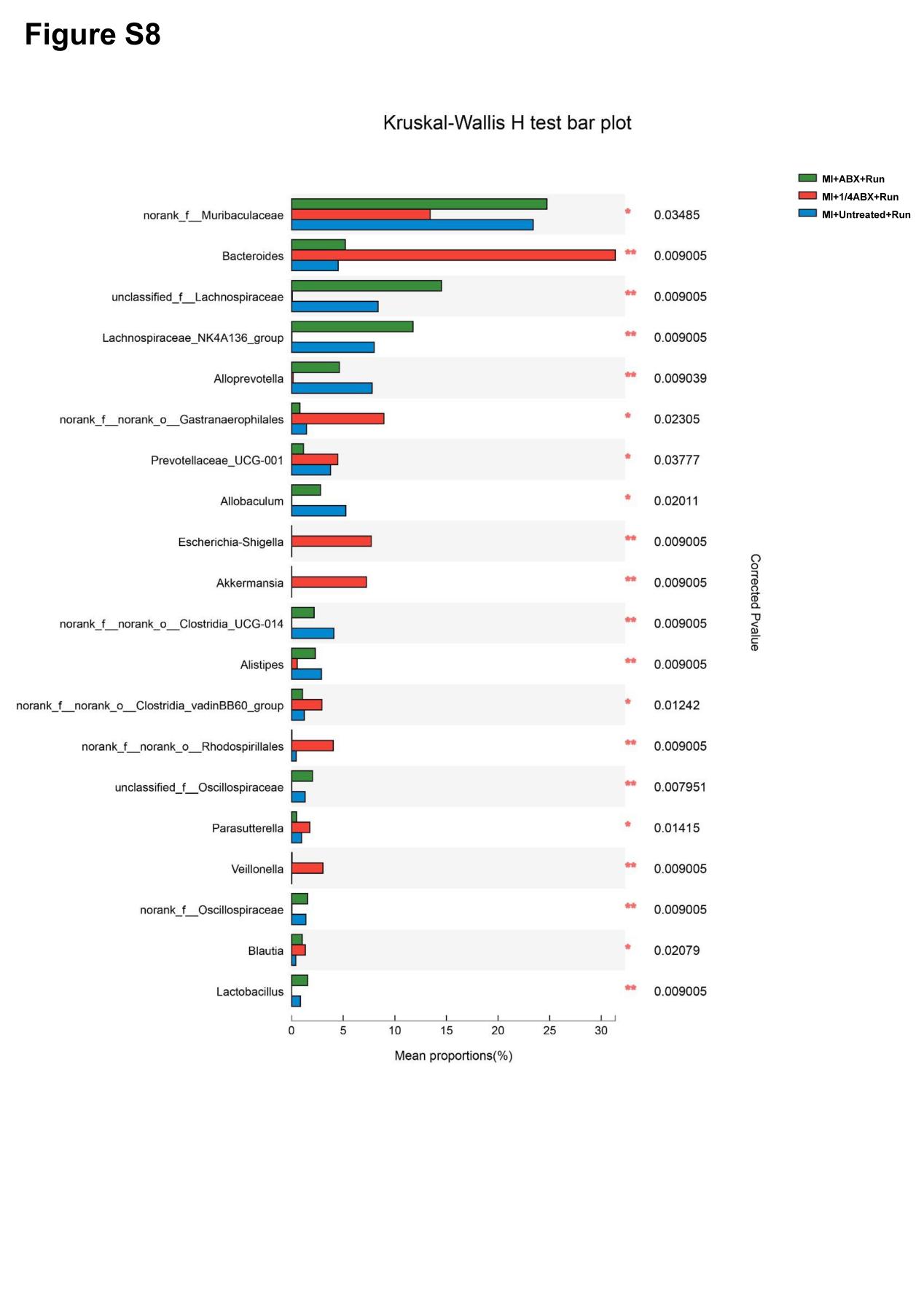


**Figure S8. Top 20 different genus across groups in the feces of mice after** **MI+Untreated+Run, MI+ABX+Run and MI+1/4ABX+Run**

Relative abundance of species across groups at the criteria of P value<0.5 by Wilcoxon rank-sum with FDR.


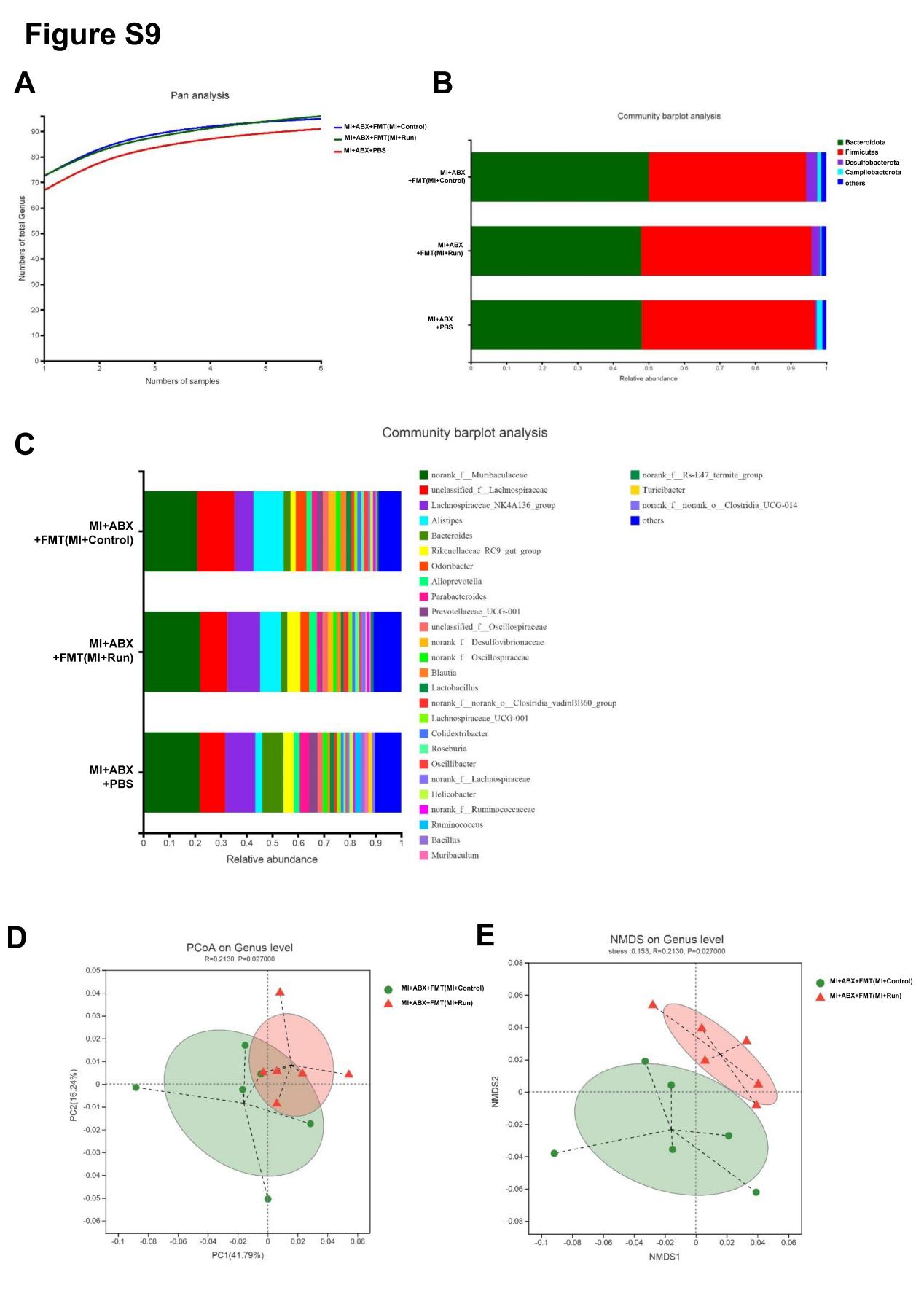


**Figure S9. Fecal microbiota transplantation (FMT) increased community richness and changed structure in mice after MI+ABX**

**A**, Pan analysis for three groups based on genus level. **B**, Fecal bacterial community at the phylum level in the feces of MI mice after FMT. **C**, Fecal bacterial community at the genus level in the feces of MI mice after FMT. **D**, PCoA analysis based on the relative abundance of genus between FMI from MI+Control and FMI from MI+Run. **E**, NMDS analysis based on the relative abundance of genus between FMI from MI+Control and FMI from MI+Run. n=6 per group; abund jaccard analysis in D and E.


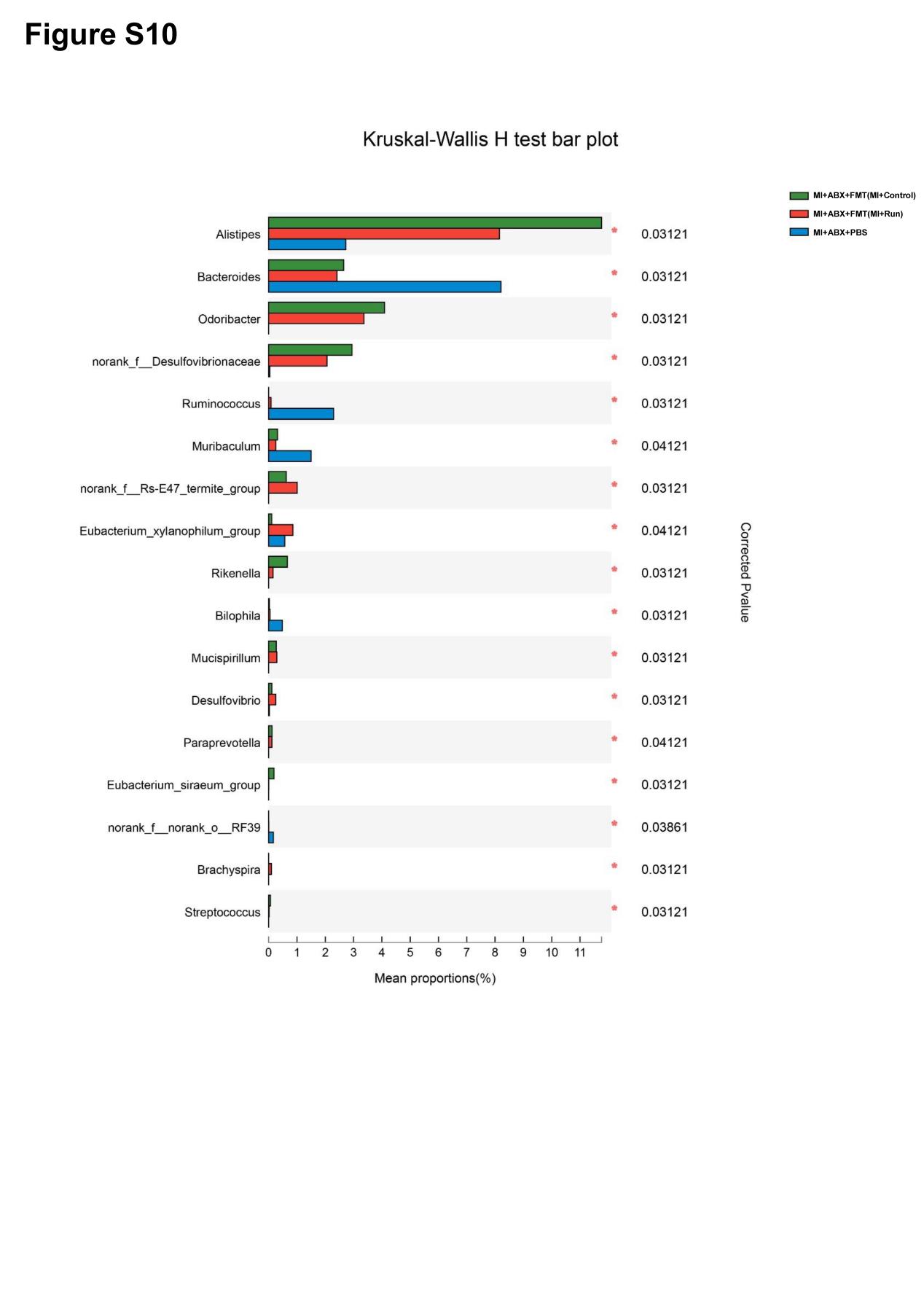


**Figure S10. Different genus across groups in the feces of mice after FMT from MI+Control, FMI from MI+Run, or without FMT (PBS)**

Relative abundance of species across groups at the criteria of P value<0.5 by Wilcoxon rank-sum with FDR.


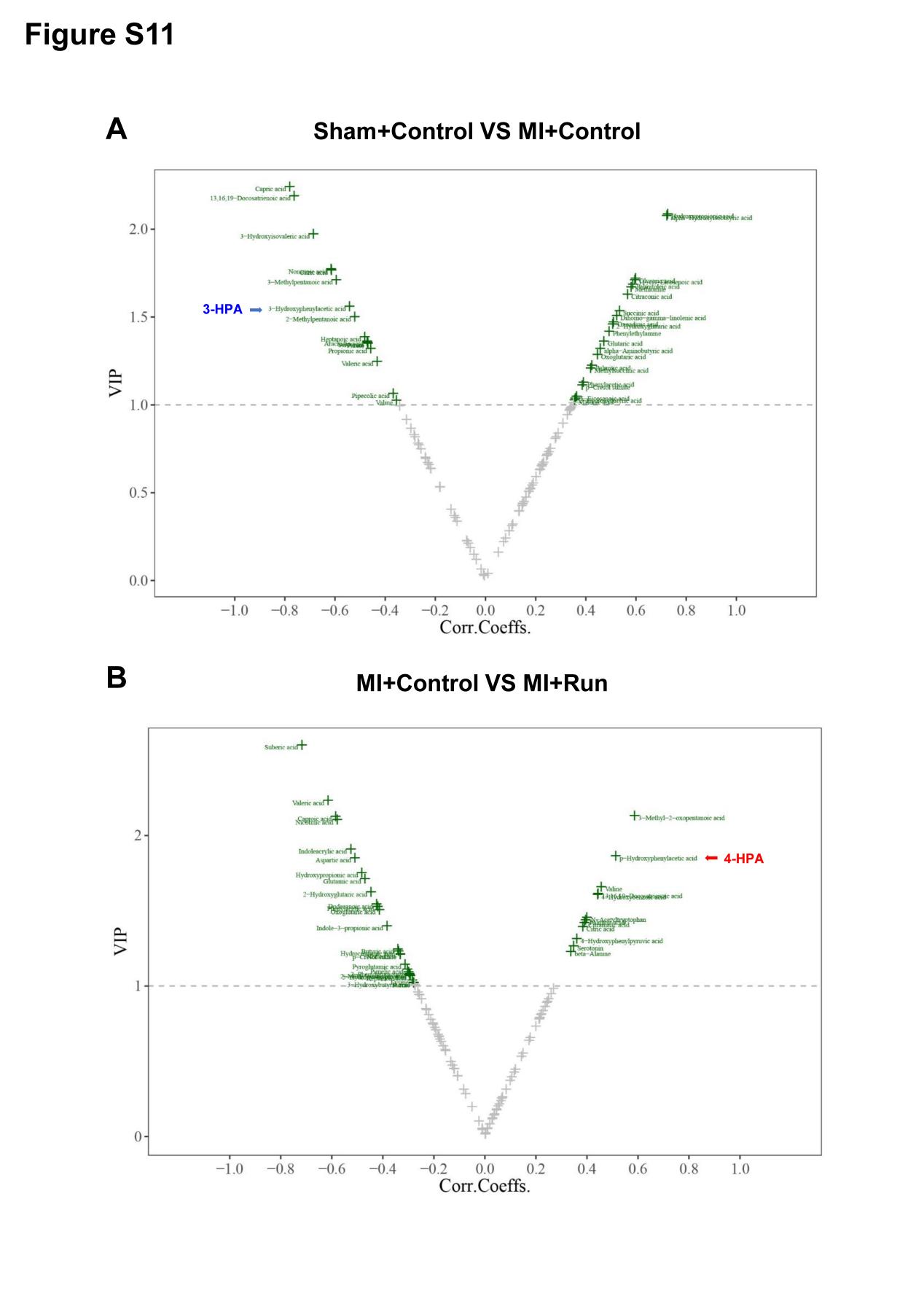


**Figure S11. 3-HPA and 4-HPA are identified by volcano plot of OPLS-DA model**

**A**, volcano plot of OPLS-DA model across Sham+Control group and MI+Control group. **B**, volcano plot of OPLS-DA model across MI+Control group and MI +Run group.


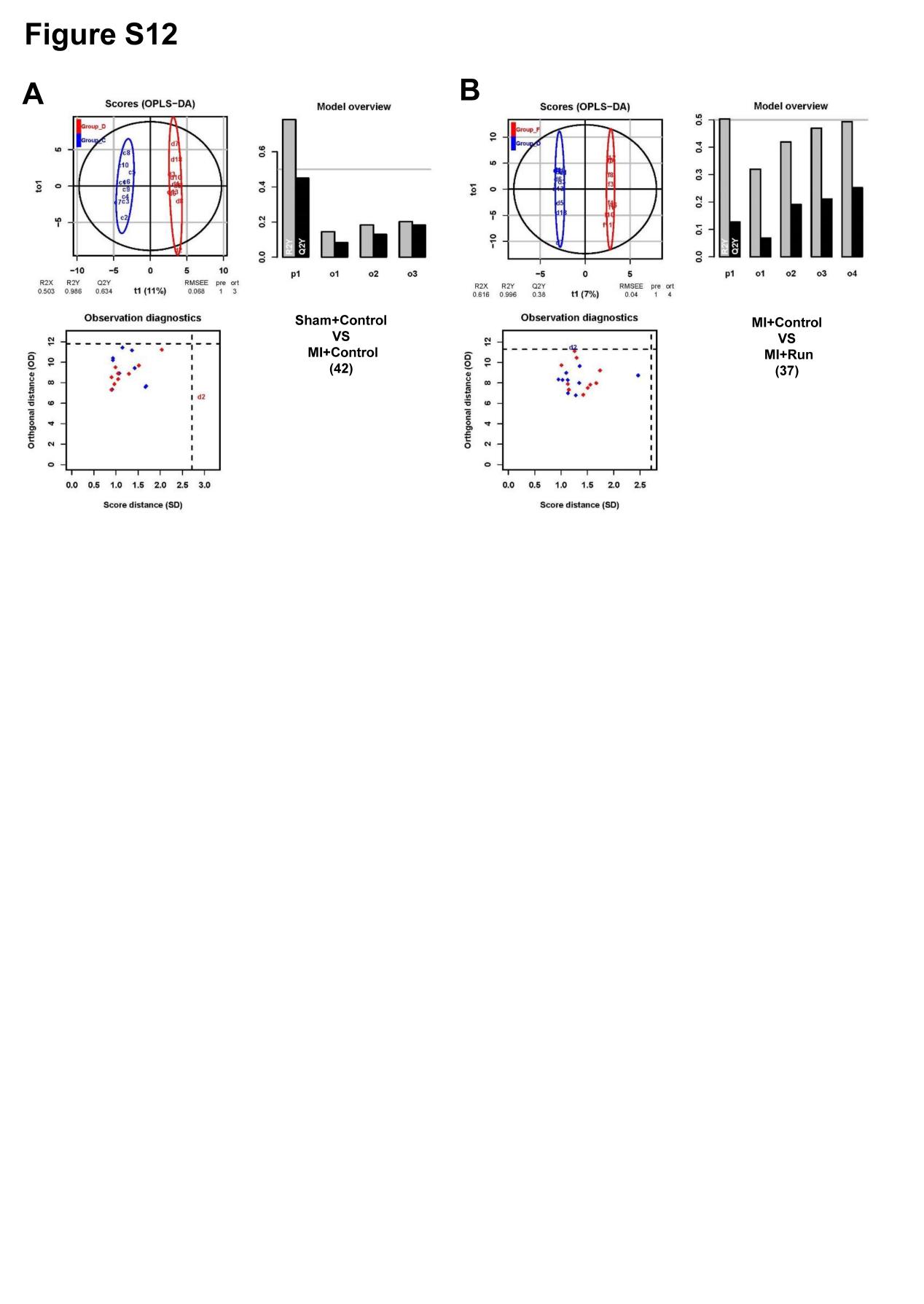


**Figure S12. 4-HBA is identified by OPLS-DA Model Discrimination**

OPLS-DA Model Discrimination based on metabolic profiles in fecal samples. **A**, Metabolic profiles of the individuals from two predefined groups, Sham+Control and MI+Control. **B**, Metabolic profiles of the individuals from two predefined groups, MI+Control vs MI+Run. The overlap of **A** and **B** was 4-HBA.

**
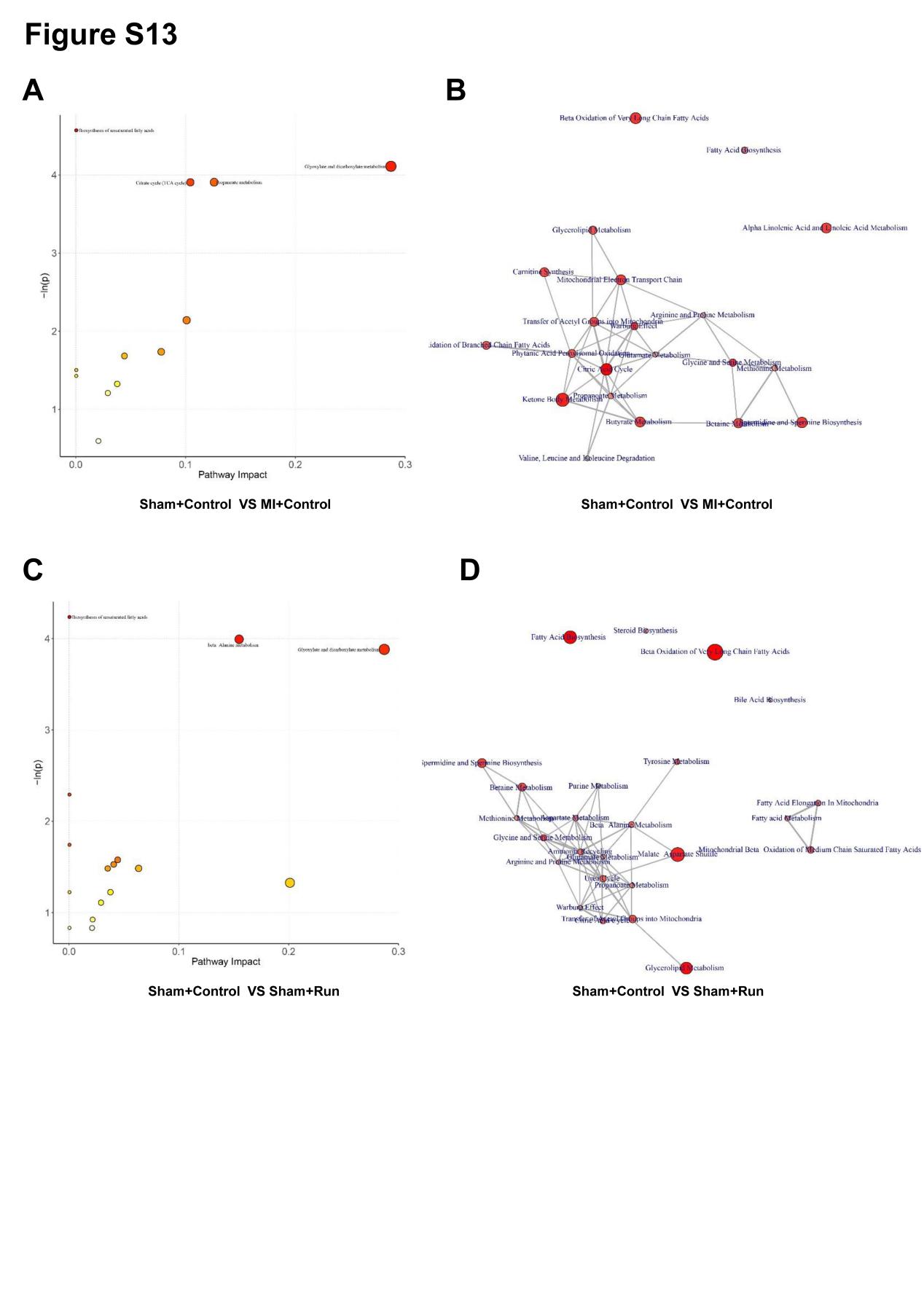
**

**Figure S13**. **Pathway analysis bubble plot and relevant network**

**A,** pathway analysis bubble plot by mmu set in Sham+Control vs MI+ Control. **B,** network for statistically significant changed pathways in Sham+Control vs MI+ Control. **C,** pathway analysis bubble plot by mmu set in Sham+Control vs Sham+Run. **D,** network for statistically significant changed pathways in Sham+Control vs Sham+Run.

**
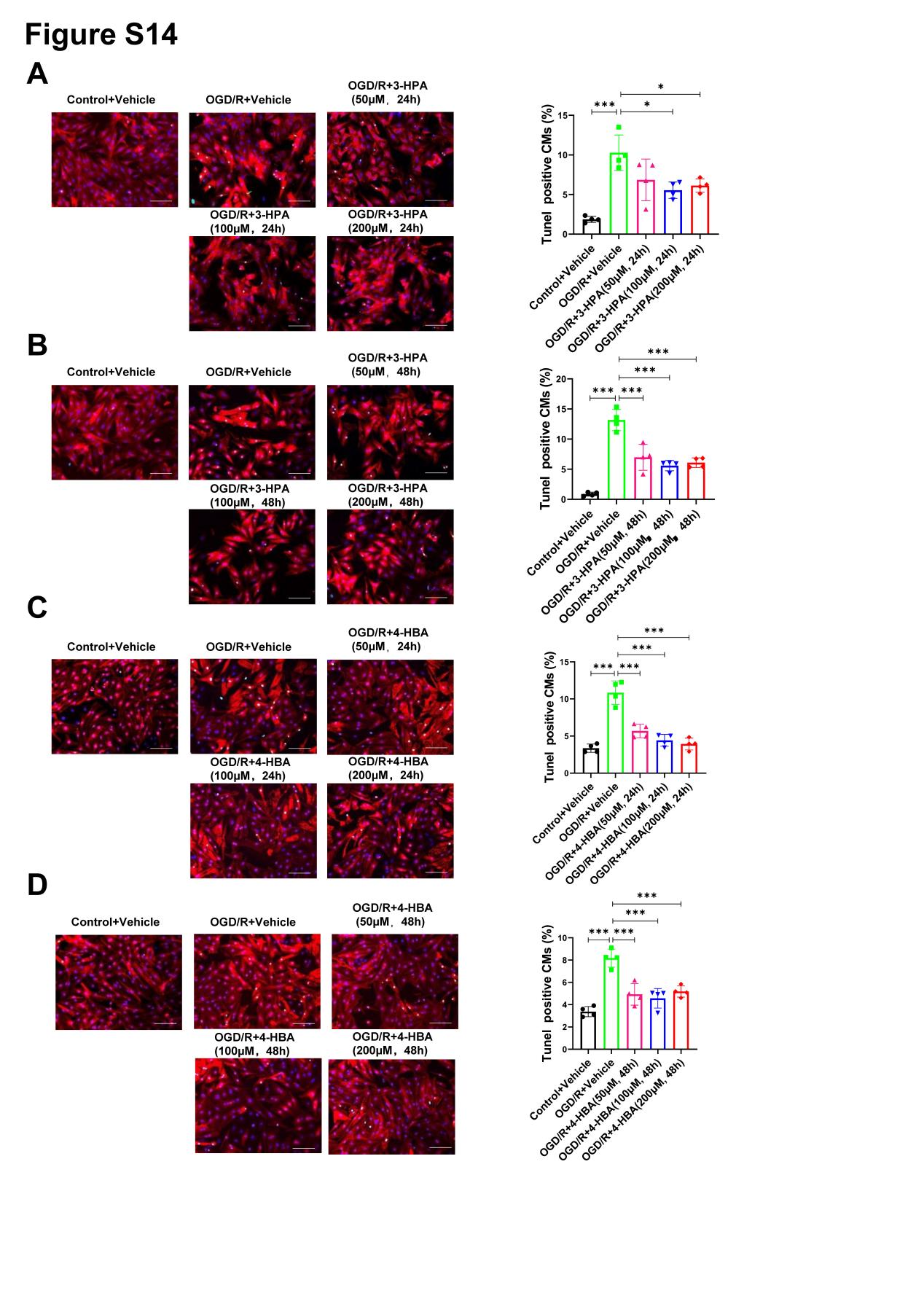
**

**Figure S14**. **3-HPA and 4-HBA decrease apoptosis with indicated duration and concentration**

3-HPA and 4-HBA decreased apoptosis induced by OGD/R in NRCMs with indicated duration and concentration (n=4 per group). Scale bar: 100μm. Data were represented as mean ± SD. Significant differences were assessed by one-way ANOVA followed by Bonferroni's multiple comparisons test. *: p<0.05, ***: p<0.001 versus respective control.
